# Novel compound heterozygous variants in the *USH2A* gene associated with autosomal recessive retinitis pigmentosa without hearing loss

**DOI:** 10.3389/fcell.2023.1129862

**Published:** 2023-02-15

**Authors:** Yanxia Huang, Lamei Yuan, Guiyun He, Yanna Cao, Xiong Deng, Hao Deng

**Affiliations:** ^1^ Health Management Center, The Third Xiangya Hospital, Central South University, Changsha, China; ^2^ Center for Experimental Medicine, The Third Xiangya Hospital, Central South University, Changsha, China; ^3^ Disease Genome Research Center, Central South University, Changsha, China; ^4^ Department of Ophthalmology, Hunan Provincial People’s Hospital, The First Affiliated Hospital of Hunan Normal University, Changsha, China; ^5^ Department of Ophthalmology, The Third Xiangya Hospital, Central South University, Changsha, China; ^6^ Department of Neurology, The Third Xiangya Hospital, Central South University, Changsha, China

**Keywords:** compound heterozygous variants, retinitis pigmentosa, usherin gene, whole exome sequencing, photoreceptor cell death

## Abstract

**Background:** Retinitis pigmentosa (RP) is a group of progressive inherited retinal dystrophies characterized by the primary degeneration of rod photoreceptors and the subsequent loss of cone photoreceptors because of cell death. It is caused by different mechanisms, including inflammation, apoptosis, necroptosis, pyroptosis, and autophagy. Variants in the usherin gene (*USH2A*) have been reported in autosomal recessive RP with or without hearing loss. In the present study, we aimed to identify causative variants in a Han-Chinese pedigree with autosomal recessive RP.

**Methods:** A six-member, three-generation Han-Chinese family with autosomal recessive RP was recruited. A full clinical examination, whole exome sequencing, and Sanger sequencing, as well as co-segregation analysis were performed.

**Results:** Three heterozygous variants in the *USH2A* gene, c.3304C>T (p.Q1102*), c.4745T>C (p.L1582P), and c.14740G>A (p.E4914K), were identified in the proband, which were inherited from parents and transmitted to the daughters. Bioinformatics analysis supported the pathogenicity of the c.3304C>T (p.Q1102*) and c.4745T>C (p.L1582P) variants.

**Conclusions:** Novel compound heterozygous variants in the *USH2A* gene, c.3304C>T (p.Q1102*) and c.4745T>C (p.L1582P), were identified as the genetic causes of autosomal recessive RP. The findings may enhance the current knowledge of the pathogenesis of *USH2A*-associated phenotypes, expand the spectrum of the *USH2A* gene variants, and contribute to improved genetic counseling, prenatal diagnosis, and disease management.

## Introduction

Retinitis pigmentosa (RP, MIM #268000) is one of the most severe hereditary retinal disorders and a major cause of visual disability and blindness (Taghipour et al., 2019; González-Del Pozo et al., 2020). It has wide phenotypic and genetic heterogeneity with numerous gene defects (Ávila-Fernández et al., 2010; Gao et al., 2019; Karali et al., 2019). The disorder is typically characterized by progressive degeneration of rod photoreceptors, cone photoreceptors, and retinal pigment epithelium, leading to night blindness, restricted peripheral vision (tunnel vision), and reduction of central vision (Hartong et al., 2006; Verbakel et al., 2018; Koyanagi et al., 2020). The estimated prevalence of the disorder is about 0.025%, and there are more than 1.5 million reported patients worldwide (de Castro-Miró et al., 2014; Menghini et al., 2020; Sharon et al., 2020). Affected individuals usually present difficulty with dark adaptation in adolescence, tunnel vision in young adulthood, and central vision loss in elder age, with a varying onset age (Jinda et al., 2014; Verbakel et al., 2018). RP may be inherited in three patterns: autosomal recessive RP (ARRP), autosomal dominant RP (ADRP), and X-linked RP (XLRP), which account for 50%–60%, 30%–40%, and 5%–15% of patients, as well as some non-Mendelian inheritance traits (Hartong et al., 2006; Ferrari et al., 2011; Chen et al., 2020; Dan et al., 2020). To date, at least 44 genes causing ARRP have been recorded in the Retinal Information Network (https://web.sph.uth.edu/RetNet/, updated on 10 January 2023) (Méndez-Vidal et al., 2013; Caruso et al., 2020; Koyanagi et al., 2020). Among them, the usherin gene (*USH2A*) was reported to be responsible for 10%–15% of ARRP. Variants in at least 23 different genes have been identified in ADRP, and there are two causative genes for XLRP (Dias et al., 2018). In the recent decade, high-throughput DNA sequencing such as the whole exome sequencing (WES) has increased the identification of the causative genes in RP patients.

In RP, early studies considered that different mechanisms were involved in photoreceptor cell death (Chang et al., 1993). Inflammation is proposed to be a result of the photoreceptor degeneration induced by the genetic defects, and meanwhile, inflammation can promote the cell death (Zhao et al., 2015). Apoptosis is known as a main mechanism for rod degeneration, and necroptosis mediates cone degeneration at later stages, which remain controversial (Murakami et al., 2012; Olivares-González et al., 2020). The nucleotide-binding oligomerization domain-like receptor family pyrin domain containing 3 (NLRP3) inflammasome activation and pyroptosis were reported in RP models (Olivares-González et al., 2020; Olivares-González et al., 2021). Excessive autophagy may induce cone cell death (Murakami et al., 2012). Although it is clear that gene variants can disrupt photoreceptor function, the exact mechanism leading to photoreceptor cell death is still poorly understood.

The present study was aimed at identifying the pathogenic variants accounting for ARRP in a Han-Chinese pedigree by WES and Sanger sequencing. The clinical characteristics and potential genetic variants were evaluated, and the novel *USH2A* compound heterozygous variants, c.3304C>T (p.Q1102*) and c.4745T>C (p.L1582P), may be responsible for ARRP phenotype in this family. Our results expand the gene variant spectrum and will serve as an efficient reference for genetic diagnostic testing for patients with suspected ARRP, which will be useful for clinical management and prognosis.

## Materials and methods

### Subjects and clinical evaluation

A three-generation Han-Chinese family with a suspected autosomal recessive inherited retinal disease was recruited for genetic analysis from Changsha, Hunan province, China. Peripheral blood samples of the family were collected from six members. Written informed consents were signed by all the enrolled individuals or guardians before their participation, and the possible consequences were explained. The study was conducted in accordance with the Declaration of the Helsinki and approved by the Institutional Review Board of the Third Xiangya Hospital, Central South University (Changsha, China). Ophthalmic evaluations of patient were comprehensively performed, including best corrected visual acuity (BCVA), intraocular pressure, slit-lamp biomicroscopy, fundus photograph, visual field, optical coherence tomography (OCT), electroretinography (ERG), and fundus fluorescein angiography (FFA). Pure-tone audiometry was carried out to determine hearing thresholds at different frequencies, and the normal hearing can be defined when the loudness is lower than 20 dB. The videonystagmography (VNG) was employed to evaluate vestibular function.

### Whole exome sequencing and bioinformatics analysis

Genomic DNA was obtained from peripheral blood samples by QIAamp DNA Mini Kit (QIAGEN, Venlo, Netherlands). Three samples of members (the patient and his parents) were submitted to KingMed Diagnostics (Changsha, China) for exome capture and sequencing. Library preparation was performed using QIAseq FX DNA Library Kit (QIAGEN), according to manufacturer’s protocols, and the targeted regions were captured by xGen Exome Research Panel v1.0 (Integrated DNA Technologies, Inc., Coralville, Iowa, United States). The sequencing was performed on an Illumina NovaSeq platform. The primary data were obtained using Bcl2fastq (v2.0.1) analysis. Trimmomatic (version 0.36) was processed to obtain the clean data by removing low quality reads, bases, adaptors, *etc.* The clean data were aligned with the human genome reference sequence (GRCh37/hg19) using the Burrows-Wheeler Aligner software, and variants were called by Genome Analysis Toolkit. The ANNOVAR tool was used to annotate and identify genetic variants. The candidate variants were filtered by the following databases, including the 1000 Genomes Project, Single Nucleotide Polymorphism database (version 154), National Heart, Lung, and Blood Institute-Exome Sequencing Project 6500, Exome Aggregation Consortium, Genome Aggregation Database, China Metabolic Analytics Project, the Human Gene Mutation Database (HGMD), and ClinVar database. Variant pathogenicity was predicted by MutationTaster2021 (https://www.genecascade.org/MutationTaster2021/), Protein Variation Effect Analyzer (PROVEAN, https://provean.jcvi.org/), the Sorting Intolerant from Tolerant (SIFT), the Polymorphism Phenotyping v2 (Polyphen-2, http://genetics.bwh.harvard.edu/pph2), and MutationAssessor (http://mutationassessor.org/r3/) (Xiang et al., 2019a; Wu et al., 2021; Xiong et al., 2021; Yu et al., 2022).

### Sanger sequencing

For the potential pathogenic variants identified by WES, Sanger sequencing was applied to test the variants in the available family members, and the segregation of candidate variants with the phenotype was analyzed in the view of inheritance pattern. Primer3 (https://primer3.ut.ee) was used to design PCR amplification primers and sequencing primers for detecting the candidate variants according to the human gene reference sequences (http://genome.ucsc.edu). Three pairs of primer sequences are as follows: 5′-TAG​CTC​CAT​TCC​AGC​AAC​CT-3′ and 5′-CAG​AGG​AAA​CCA​CAA​CAG​CA-3′, 5′-TTC​GAA​CAA​AAG​TGC​CTG​AA-3′ and 5′-AGC​TGA​GGG​CAA​GTC​ACA​TT-3′, 5′-ACT​CAG​CCC​TCC​CCT​GTA​CT-3′ and 5′-AGT​GGC​TTC​TCC​GAG​TTT​CA-3′. The Chromas software (v2.01, Technelysium Pty Ltd., South Brisbane, Australia) was applied to analyze the sequencing results.

### Variant evaluation, conservative analysis, and structure modeling

The American College of Medical Genetics and Genomics (ACMG) guidelines for variant interpretation were utilized following the terms, “pathogenic”, “likely pathogenic”, “uncertain significance”, “likely benign”, and “benign” (Richards et al., 2015). Multiple sequence alignments for eleven species were conducted using Basic Local Alignment Search Tool of National Center for Biotechnology Information (https://blast.ncbi.nlm.nih.gov/Blast.cgi). The detailed information of protein was accessed from the Universal Protein Resource (UniProt, https://www.uniprot.org/). The wild-type and variant protein structures were predicted by the online CPHmodels-3.2 (https://services.healthtech.dtu.dk/service.php?CPHmodels-3.2). The three-dimensional structures were shown by PyMOL software (v2.3, Schrödinger, Inc., New York, United States) (Xiang et al., 2019b; Wu et al., 2019; Huang et al., 2021; Yuan et al., 2021).

## Results

### Clinical data

**FIGURE 1:**
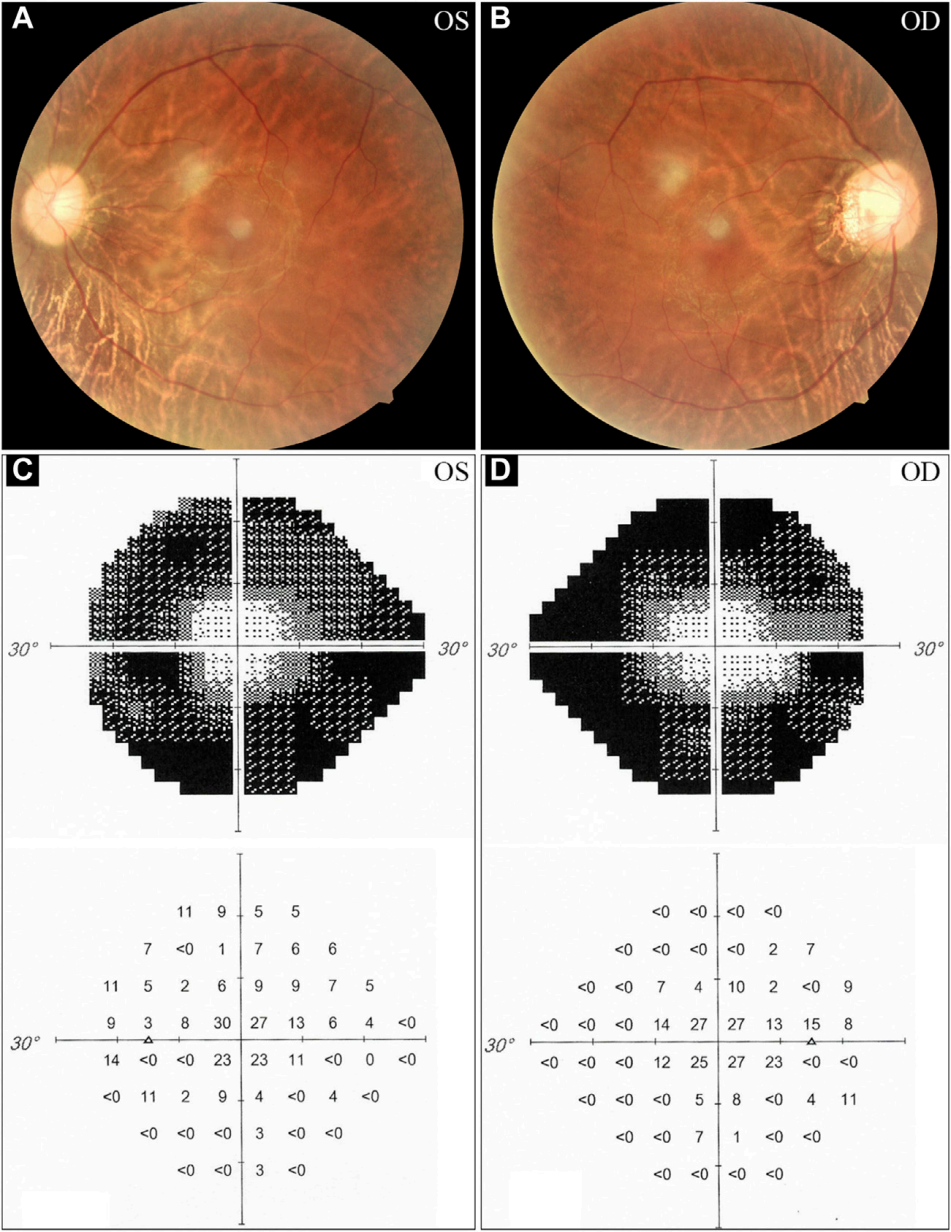
Fundus photographs and visual field tests from the proband. **(A, B)** The fundus examinations demonstrate pigmentary deposits, attenuated vessels, and waxy optic discs. **(C, D)** Visual field assessments reveal peripheral vision loss in the proband. OS, oculus sinister (left eye); OD, oculus dexter (right eye).

**FIGURE 2:**
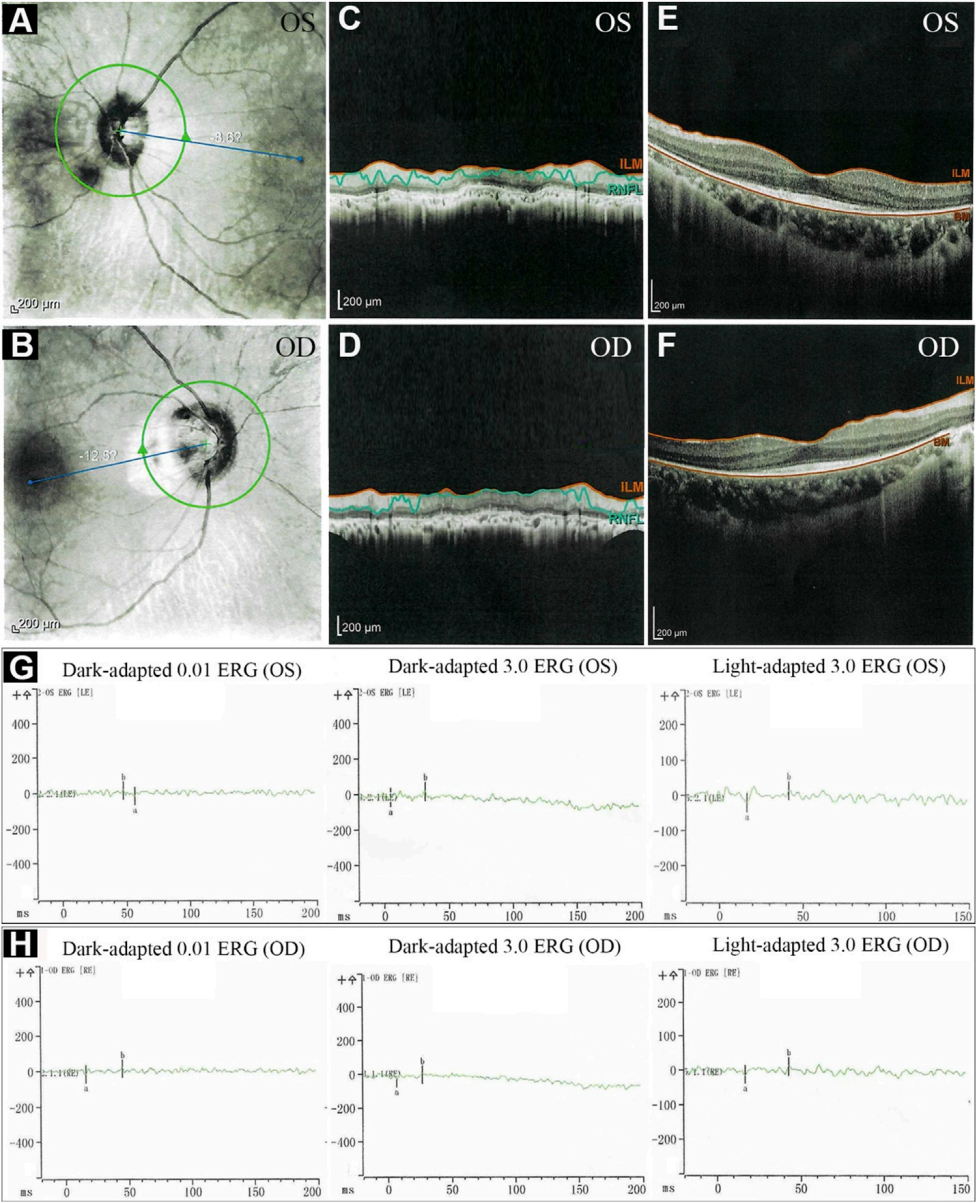
The optical coherence tomography (OCT) and electroretinography (ERG) of the proband. **(A–F)** OCT showed thinning of retinal nerve fiber layer. **(G, H)** ERG showed significantly reduced scotopic and photopic responses, with extinguished decrease of a and b waves. ILM, internal limiting membrane; RNFL, retinal nerve fiber layer; BM, Bruch’s membrane; OS, oculus sinister (left eye); OD, oculus dexter (right eye).

**FIGURE 3:**
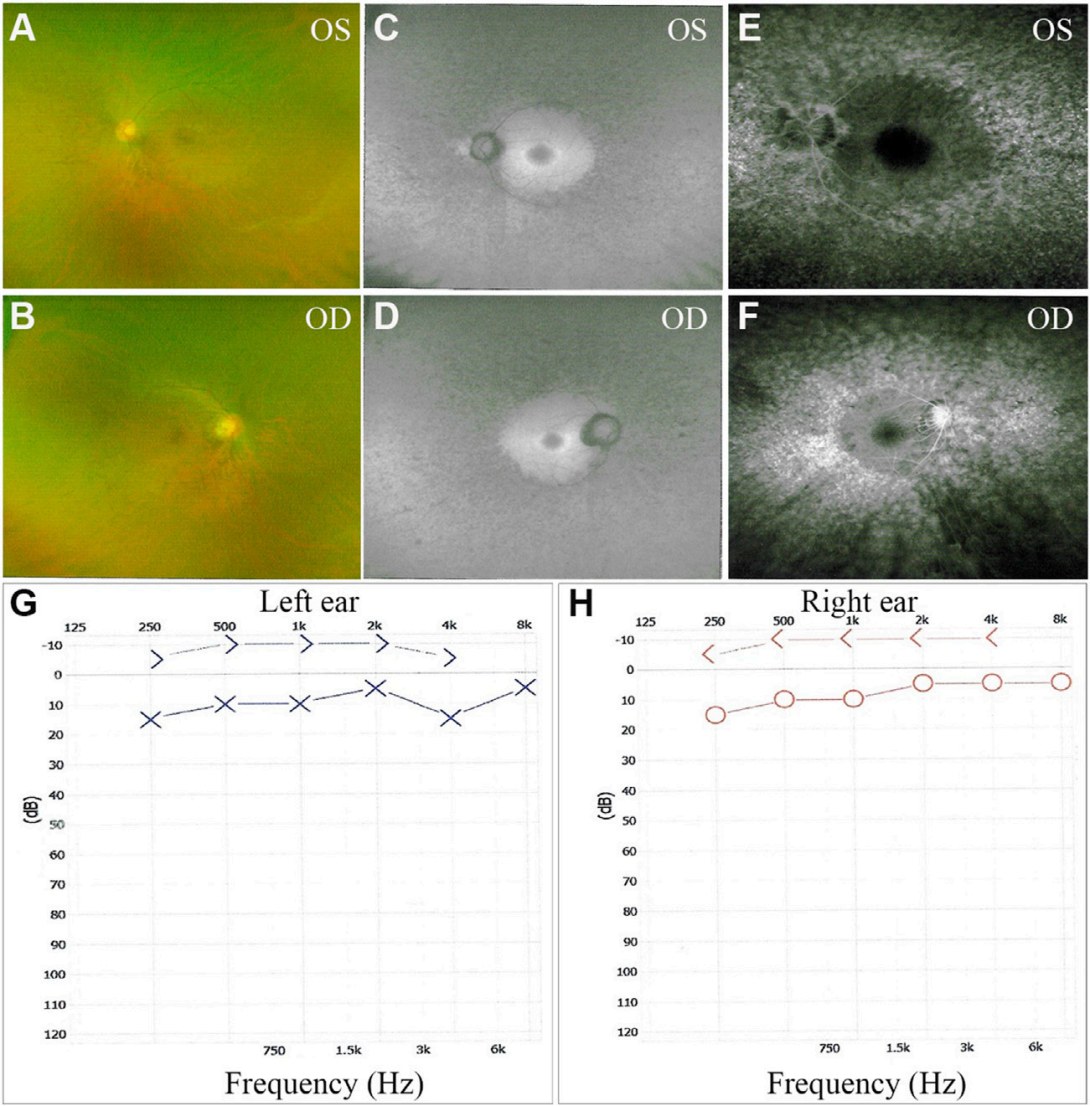
Fundus fluorescein angiography (FFA) and pure-tone audiograms of the proband. **(A–F)** Bilateral fluorescein leakage on FFA. **(G, H)** Audiograms showed no hearing loss in both ears. OS, oculus sinister (left eye); OD, oculus dexter (right eye).

The proband, a 34-year-old male, complained of night blindness, constricted vision field, and reduced vision. The BCVA test results were 0.6 in both eyes. The intraocular pressure and anterior segment examination were normal. The fundus photographs revealed waxy-pale appearance of the discs, attenuated retinal arteries, and pigment deposits (Figures 1A,B). The visual field test indicated peripheral vision loss in both eyes (Figures 1C,D). The OCT revealed that the retinal nerve fiber layer was markedly thinned (Figures 2A–F). Full-field ERG showed significantly decreased scotopic and photopic responses, with extinguished decrease of a and b waves (Figures 2G,H). FFA showed hyperfluorescence and hypofluorescence areas and attenuated vessels in both eyes (Figures 3A–F). Hearing examination was normal by pure-tone audiometry test (Figures 3G,H). Vestibular assessments showed intact vestibular function according to the VNG test (Supplementary Table S1). The patient matched the clinical diagnostic criteria of RP. Parental consanguineous marriage was denied.

### Whole exome sequencing and identification of pathogenic variants

**FIGURE 4:**
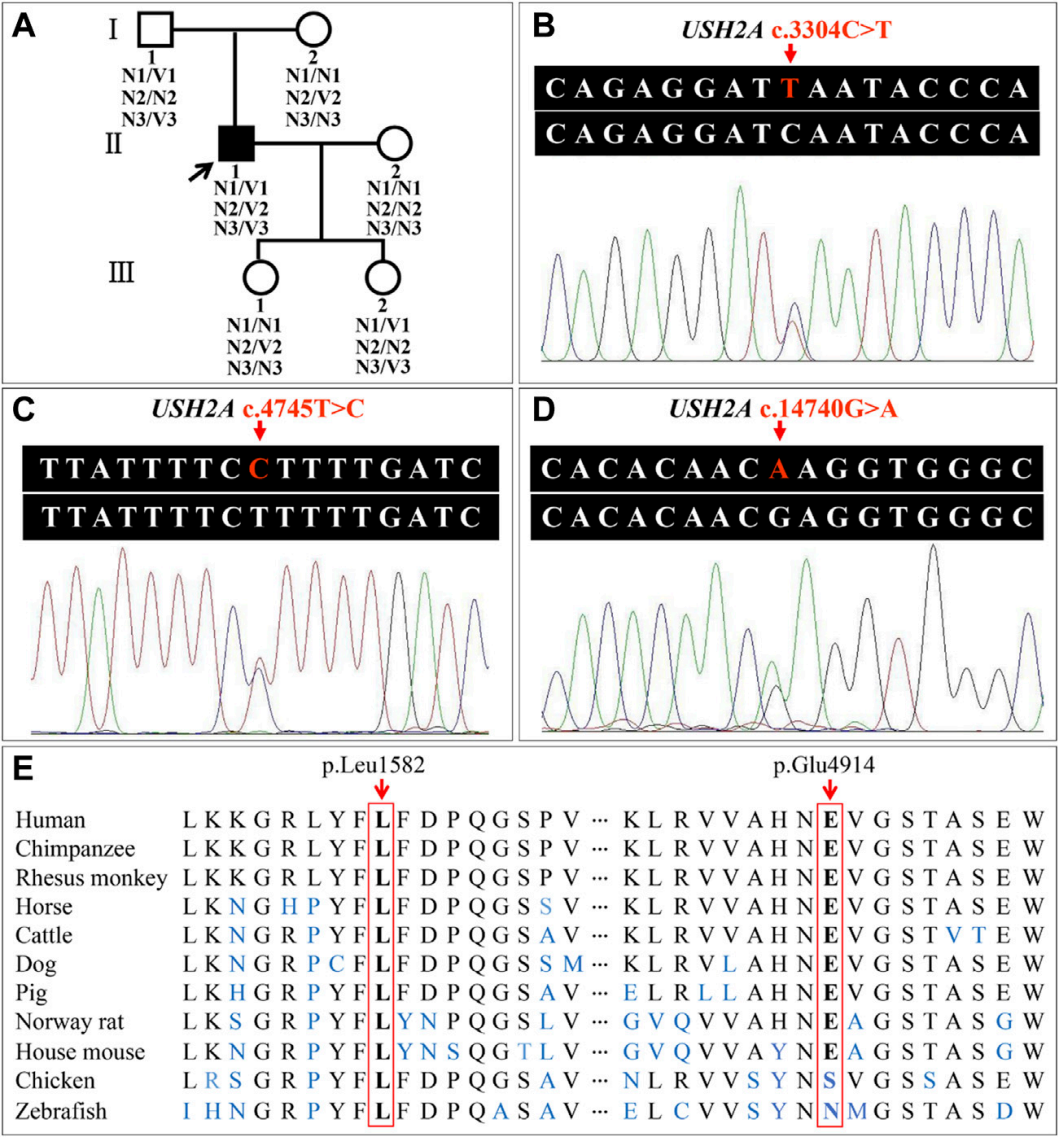
Pedigree of the family with autosomal recessive retinitis pigmentosa and analysis of the usherin gene (*USH2A*) variants. **(A)** Pedigree figure. The squares and the circles symbolize males and females, respectively. Filled symbol represents the patient, and empty symbols represent unaffected members. The arrow indicates the proband. N1, allele with *USH2A* c.3304C; V1, allele with *USH2A* c.3304T; N2, allele with *USH2A* c.4745T; V2, allele with *USH2A* c.4745C; N3, allele with *USH2A* c.14740G; V3, allele with *USH2A* c.14740A. **(B–D)** Patient (II:1) with variants c.3304C>T, c.4745T>C, and c.14740G>A in the *USH2A* gene. **(E)** Conservation analysis of the USH2A p.Leu1582 and p.Glu4914 residues.

**FIGURE 5:**
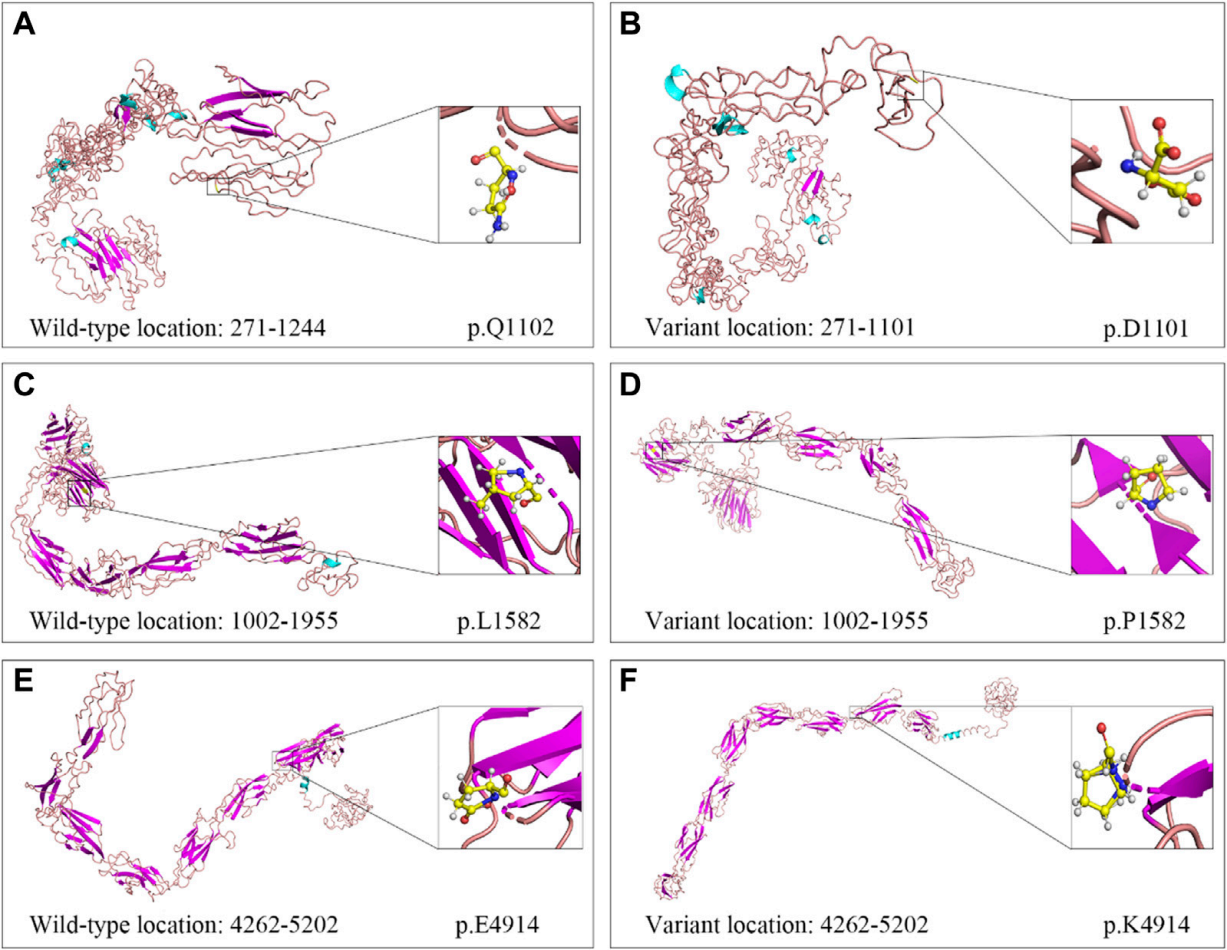
Cartoon models of wild-type and variant USH2A protein in three regions. **(A, B)** Segment 1 (amino acid 271–1,244): c.3304C>T (p.Q1102*). **(C, D)** Segment 2 (amino acid 1,002–1,955): c.4745T>C (p.L1582P). **(E, F)** Segment 3 (amino acid 4,262–5,202): c.14740G>A (p.E4914K).

WES of three individuals (I:1, I:2, and II:1, Figure 4A) generated a total of 239.41 million (84.24 million, 73.67 million, and 81.50 million) clean reads, and the target region with a mean coverage of 99.86% (99.91%, 99.74%, and 99.92%) was mapped to the human reference genome. The mean depth of target region was 162.72-fold (174.23-fold, 149.84-fold, and 164.08-fold). A total of 51,803 single nucleotide polymorphisms (SNPs) and 8,129 insertions-deletions (indels) were found in the father (I:1), and a total of 52,281 SNPs and 8,028 indels were found in the mother (I:2). There were a total of 51,919 SNPs and 8,042 indels in the proband (II:1). Three heterozygous variants (c.3304C>T, p.Q1102*; c.4745T>C, p.L1582P; c.14740G>A, p.E4914K) in the *USH2A* gene (NM_206933.4), a causative gene for ARRP, were identified in the proband. The two variants c.3304C>T and c.14740G>A were inherited from his father, and the c.4745T>C variant was originated from his mother. The nonsense variant, c.3304C>T (p.Q1102*), was predicted to be deleterious by MutationTaster2021. The missense variant, c.4745T>C (p.L1582P), was predicted to be deleterious by MutationTaster2021, PROVEAN, SIFT, and PolyPhen-2. The c.14740G>A (p.E4914K) variant was predicted to be benign (Table 1). Sanger sequencing confirmed the heterozygous variants in the five family members (Figures 4A–D). Co-segregation analysis supported that compound heterozygous variants (c.3304C>T and c.4745T>C) were responsible for the disease phenotype. The c.3304C>T variant was classified as “likely pathogenic” variant, and the c.4745T>C and c.14740G>A variants were classified as “uncertain significance” variants according to the comprehensive analysis of ACMG guidelines. Conservative analysis of the USH2A protein from zebrafish to human showed that leucine at variant site (p.Leu1582) was highly conserved and glutamic acid at variant site (p.Glu4914) was conserved from house mouse to human (Figure 4E). Structural modeling showed the conformational alteration of the protein (UniProt Knowledgebase: O75445, Figure 5).

**TABLE 1:** Identification of *USH2A* variants in the proband.

Category	Variant 1	Variant 2	Variant 3
Nucleotide change	c.3304C>T	c.4745T>C	c.14740G>A
Amino acid change	p.Q1102*	p.L1582P	p.E4914K
Exon	16	22	67
Zygosity	Heterozygote	Heterozygote	Heterozygote
Variant type	Nonsense	Missense	Missense
1000G	No	No	2.00 × 10^−4^
dbSNP154	No	No	rs199829169
NHLBI-ESP6500	No	No	No
ExAC	No	No	9.89 × 10^−5^
gnomAD	No	No	9.55 × 10^−5^
ChinaMAP	No	No	1.46 × 10^−3^
HGMD	No	No	No
ClinVar	No	No	Likely benign
MutationTaster2021	Deleterious	Deleterious	Benign
PROVEAN	NA	Deleterious	Neutral
SIFT	NA	Damaging	Tolerated
PolyPhen-2	NA	Probably damaging	Benign
MutationAssessor	NA	Medium	Low

1000G, 1000 Genomes Project; dbSNP154, Single Nucleotide Polymorphism database version 154; NHLBI-ESP6500, National Heart, Lung, and Blood Institute-Exome Sequencing Project 6500; ExAC, Exome Aggregation Consortium; gnomAD, Genome Aggregation Database; ChinaMAP, China Metabolic Analytics Project; HGMD, Human Gene Mutation Database; PROVEAN, Protein Variation Effect Analyzer; SIFT, Sorting Intolerant from Tolerant; PolyPhen-2, Polymorphism Phenotyping v2; NA, not applicable.

## Discussion

In 1998, three biologically important variants (c.2299del, c.2898del, and c.4336_4337del) in the *USH2A* gene were reported to be responsible for the autosomal recessive disorder, Usher syndrome type IIA (USH2A, MIM #276901), featured by sensorineural hearing impairment and progressive RP (Eudy et al., 1998; McGee et al., 2010). Subsequently, the *USH2A* missense variant p.C759F was firstly reported to be associated with ARRP without hearing impairment in 2000, which was presumed to affect the disulfide bridge in the fifth laminin epidermal growth factor-like domain of the USH2A protein (Rivolta et al., 2000; Lenassi et al., 2015). Until now, over 1,155 likely pathogenic variants in the *USH2A* gene have been recorded in HGMD, responsible for non-syndromic and syndromic RP (i.e., ARRP and USH2A). The *USH2A* gene variants were reported to be the most common cause (29%) of Usher syndrome and one of the most common causes (19%–23%) of ARRP (Gao et al., 2019; Zhu et al., 2020).

In this study, novel compound heterozygous variants in the *USH2A* gene, c.3304C>T (p.Q1102*) and c.4745T>C (p.L1582P), were prosecuted as disease causative factors for a Han-Chinese pedigree with RP by a full ophthalmological examination, WES, bioinformatics analysis, Sanger sequencing, and co-segregation analysis. These two variants were predicted to be harmful by several prediction softwares.

The *USH2A* gene, located on chromosome 1q41, has two transcripts due to alternatively splicing (21 exons and 72 exons), encoding the secreted and transmembrane proteins, respectively (Inaba et al., 2020; Meng et al., 2021). The longer isoform is a transmembrane protein of 5,202 amino acids (∼600 kDa), which is a cilial protein and mainly expressed in the connecting cilia of the photoreceptors and developing inner ear hair cells (Méndez-Vidal et al., 2013; Huang et al., 2018; Castiglione and Möller, 2022). The USH2A plays an essential role in the homeostasis, development, and function of visual and auditory sensory (Molina-Ramírez et al., 2020; Toms et al., 2020). The protein includes three regions: i) a large extracellular region with a signal peptide, a laminin N-terminal, 10 laminin epidermal growth factor-like domains, and 34 fibronectin type-III domains separated by 2 laminin G-like domains; ii) a transmembrane region; iii) a short intracellular region with a PDZ-binding motif at its C-terminal end (Liu et al., 2007; Chen et al., 2014; Fu et al., 2020; Zhu et al., 2021). The *USH2A* variants, including missense, nonsense, duplications/insertions, deletions, indels, and splicing variants, spread throughout the 72 exons and their flanking intronic regions (Aller et al., 2006; Koyanagi et al., 2020; Gao et al., 2021). Genotype-phenotype correlation has yet to be elucidated, while analyses based on some studies showed that patients with truncating variants may have more severe phenotypes, involving visual and auditory dysfunction (Inaba et al., 2020; Meng et al., 2021). Non-syndromic RP (typical or isolated RP) may be caused by variants in genes related to retina-specific expressions or functions, whereas syndromic RP (RP with extra-ocular defects) may be aroused by variants in genes functioning in various cells or tissues (Dias et al., 2018; Verbakel et al., 2018). However, the precise mechanisms of those variants underlying RP and Usher syndrome are still unclear.

**FIGURE 6:**
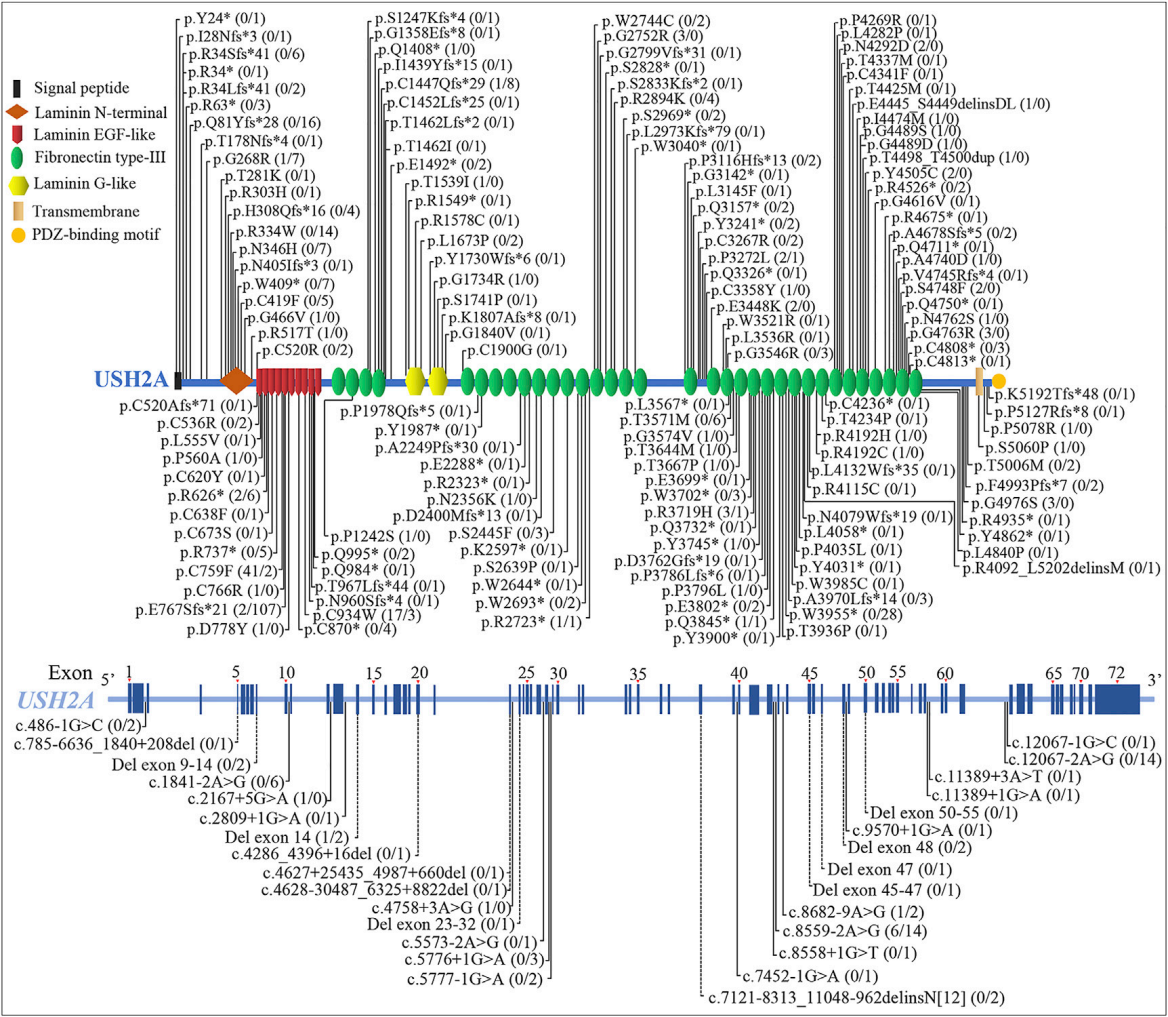
Schematic distribution of the usherin gene (*USH2A*) homozygous variants associated with retinitis pigmentosa (RP) and Usher syndrome type IIA (USH2A). Numbers in parentheses after the variants represent the number of patients with RP and USH2A, separated by the slash. The dotted line indicates deletion or deletion-insertion with changes over 20 bp. EGF, epidermal growth factor.

**FIGURE 7:**
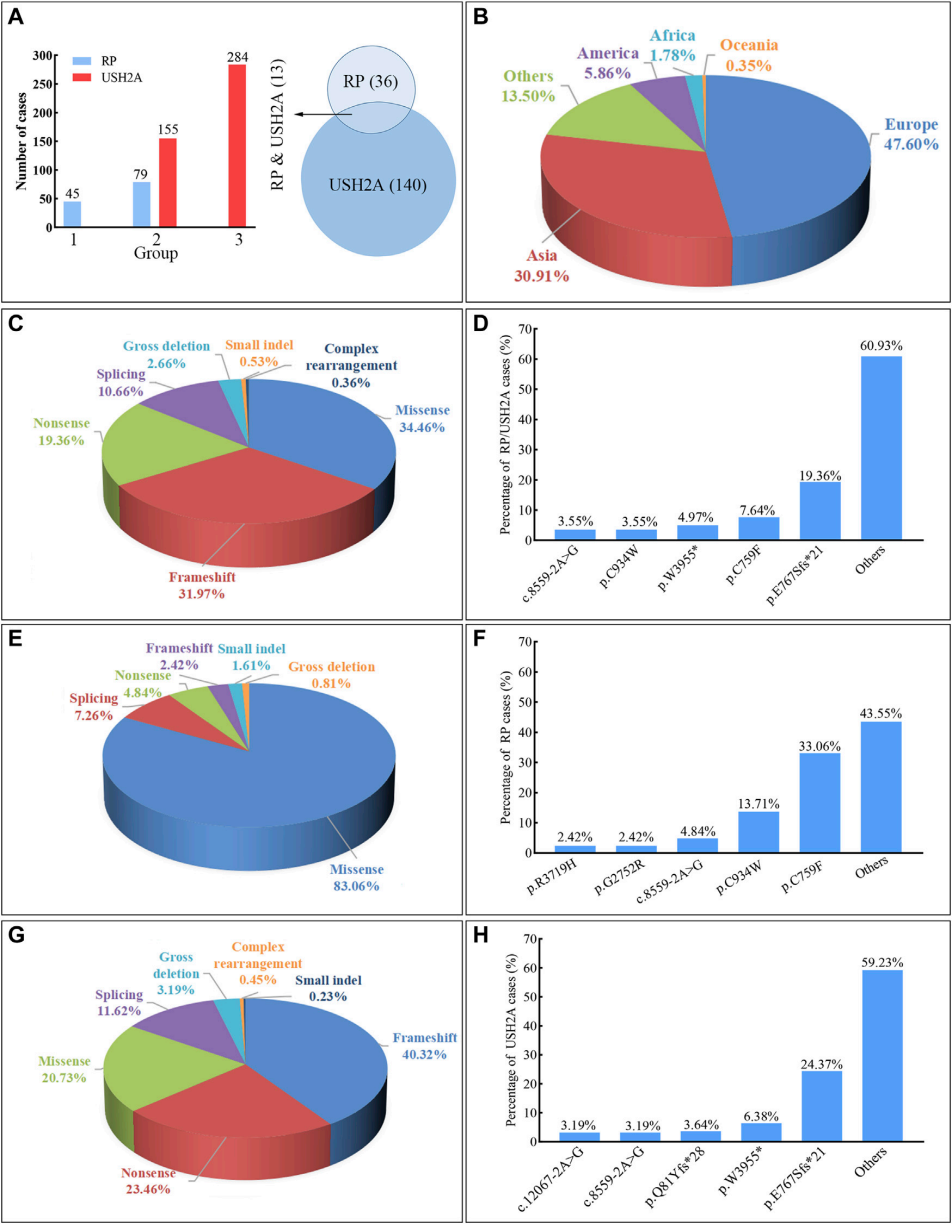
Summary of the usherin gene (*USH2A*) homozygous variants associated with retinitis pigmentosa (RP) and Usher syndrome type IIA (USH2A). Frameshift includes small deletion and duplication/insertion with changes involving 20 bp or less leading to reading frame shift. Small indel includes duplication and deletion-insertion with changes involving 20 bp or less leading to one or more amino acids inserted or replaced. Gross deletion or complex rearrangement (i.e., complex deletion-insertion) refers to changes over 20 bp. **(A)** The bar chart indicates the case number of RP or USH2A caused by various variants, in which the group “1” represents the variant causing RP, the group “2” represents the variant causing both RP and USH2A, and the group “3” represents the variant causing USH2A. The Venn diagram shows the reported numbers of variants causing RP and USH2A. **(B)** The proportion of RP and USH2A patients carrying the *USH2A* homozygous variants in different continents. **(C)** The proportion of cases with RP and USH2A by different variant types. **(D)** The percentage of cases with RP and USH2A observed at different variants. **(E)** The proportion of cases with RP caused by different variant types. **(F)** The percentage of cases with RP observed at different variants. **(G)** The proportion of cases with USH2A by different variant types. **(H)** The percentage of cases with USH2A observed at different variants.

At least 189 homozygous *USH2A* pathogenic variants were observed in 563 patients (RP and USH2A) from different populations in the worldwide (Figures 6, 7, and Supplementary Table S2), including European (47.60%, 268/563), Asian (30.91%, 174/563), American (5.86%, 33/563), African (1.78%, 10/563), and Oceanian (0.35%, 2/563). The most frequent homozygous variants in RP patients are missense variants, in which p.C759F and p.C934W account for 46.77% (58/124). Five most frequent variants in USH2A patients are p.E767Sfs*21 (24.37%, 107/439), p.W3955* (6.38%, 28/439), p.Q81Yfs*28 (3.64%, 16/439), c.8559–2A>G (3.19%, 14/439), and c.12067–2A>G (3.19%, 14/439). The common homozygous missense variant p.C759F is liable to lead to RP, while the homozygous c.2299del (p.E767Sfs*21) variant tends to cause USH2A. The extra-, even intra-familial clinical differences including penetrance and manifestations related to the same variant suggest that genetic background, epigenetics, and the environmental factors might influence the phenotypes.

Homozygous *Ush2a* knockout mice and zebrafish have progressive photoreceptor degeneration and non-progressive hearing impairment, similar to the visual and auditory deficits in human patients with *USH2A* variants. Two mutant *ush2a* zebrafish with different domains ablated had early-onset retinal dysfunction aggravated by sustained light exposure, indicating the critical role of the protein in maintenance of photoreceptors and development of hair cells, especially the intracellular domain in visual function (Dona et al., 2018; Han et al., 2018).

RP is caused by different cell death pathways and mechanisms. Some studies showed that necroptosis was implicated in both rod and cone cell death in animal models, and upregulation of receptor-interacting protein 1 and 3 kinase complexes was related with the necroptosis in photoreceptors (Murakami et al., 2012; Sato et al., 2013; Viringipurampeer et al., 2016; Viringipurampeer et al., 2019). The high expression level of tumor necrosis factor alpha and oxidative stress could induce the activation of NLRP3 inflammasome, including NLRP3, caspase-1, and interleukin 1 beta expression, and precipitate pyroptosis in rd10 mouse retinas at postnatal day 23 (Olivares-González et al., 2020). Autophagy was found to be involved in the photoreceptor cell death in some RP models, and the inhibition could exert a protective role (Rodríguez-Muela et al., 2015; Yao et al., 2018; Qiu et al., 2019). These studies demonstrate that more than one cellular mechanism is activated in retinal degeneration, which may be genotype-specific. Therefore, further research on the mechanisms of rod and cone photoreceptor cell death would be beneficial for the potential treatment of RP.

Given that patients may miss or refuse hearing examinations, the diagnosis of USH2A may be omitted in some cases, and the true frequency of *USH2A*-associated ARRP may be lower. In this study, pure-tone audiometry and vestibular examination were conducted, and the hearing acuity of the proband fell within the normal range, indicating the compound heterozygous *USH2A* variants may cause ARRP alone.

In conclusion, novel compound heterozygous variants, c.3304C>T (p.Q1102*) and c.4745T>C (p.L1582P), were identified as the genetic causes for ARRP in a Han-Chinese family by WES and Sanger sequencing. Our study expands the spectrum of the *USH2A* gene variants and may contribute to improved genetic counseling, prenatal diagnosis, and disease management for this family. Further construction of site-specific gene-deficient animal models and functional study may help to understand the genetic mechanism of *USH2A*-associated ARRP, which may further provide a clue to develop target therapy of this complex disorder.

## Data Availability

Data of this project can be accessed after an approval application by the China National GeneBank DataBase (CNGBdb). Please refer to https://db.cngb.org/, or email: CNGBdb@cngb.org for detailed application guidance. The project accession code CNP0003957 should be included in the application.
