# Supplementary material for: Novel compound heterozygous variants in the USH2A gene associated with autosomal recessive retinitis pigmentosa without hearing loss
Source: Front Cell Dev Biol. 2023 Feb 15;11:1129862. doi: 10.3389/fcell.2023.1129862 (PMC9974670; doi:10.3389/fcell.2023.1129862)
Supplement: Supplementary file 1 [file DataSheet1.pdf]

Supplementary Material

1 Supplementary Tables

Supplementary Table S1 Videonystagmography in the proband.

Supplementary Table S1A Eye movement test.

Category	Result
Spontaneous nystagmus	No
Gaze test	Normal
Smooth pursuit test	Normal
Saccade test	Normal
Optokinetic test	Normal

Supplementary Table S1B Positional nystagmus test.

Position	Result
Supine head center	-
Head right	-
Head left	-
Body right	-
Body left	-

Supplementary Table S1C Positioning nystagmus test.

Position	Dix-Hallpike test	Roll test
Head-hanging left	-	NA
Head-hanging right	-	NA
Head-turning left	NA	-
Head-turning right	NA	-

Supplementary Table S1D Vestibular caloric test.

Ear	Temperature (℃)	Fixation suppression index[a]	Slow phase velocity (°/s)	Unilateral weakness[b]	Directional preponderance[c]
Right	24	100%	35.7	NA	14%
	49	100%	71.5		
Left	24	96%	42.2	7%	NA
	49	100%	50.7		

NA, not applicable; -, negative.

[a] Normal reference range for fixation suppression index <60%; [b] Unilateral weakness = [(SPV in R49 + SPV in R24) - (SPV in L49 + SPV in L24)] / (SPV in R49 + SPV in R24 + SPV in L49 + SPV in L24), normal reference range <25%; [c] Directional preponderance = [(SPV in R49 + SPV in L24) - (SPV in L49 + SPV in R24)] / (SPV in R49 + SPV in R24 + SPV in L49 + SPV in L24), normal reference range <30%. SPV represents slow phase velocity, R represents right ear, and L represents left ear.

The abnormal fixation suppression index may be related to the proband's retinitis pigmentosa, and the fixation point can be lost with the eye patch.

Supplementary Table S2 The reported RP and USH2A patients with the homozygous *USH2A* variants.

No.	Number of patients	Phenotype	Nucleotide change[a]	Amino acid change[a]	Variant type[b]	Exon/ Intron[c]	Domain	Geographical/ Ethnic distribution	Continent	Reference
1	1	USH2A	c.72T>G	p.Y24*	Nonsense	E2	SP	European	Europe	Baux et al., 2014
2	1	USH2A	c.83del	p.I28Nfs*3	Frameshift	E2	SP	European	Europe	Baux et al., 2014
3	1	USH2A	c.99_100insT	p.R34Sfs*41	Frameshift	E2	–	Chinese	Asia	Sun et al., 2018
4	1	USH2A	c.99_100insT	p.R34Sfs*41	Frameshift	E2	–	Chinese	Asia	Fu et al., 2020
5	3	USH2A	c.99_100insT	p.R34Sfs*41	Frameshift	E2	–	Chinese	Asia	Gao et al., 2021
6	1	USH2A	c.99_100insT	p.R34Sfs*41	Frameshift	E2	–	Chinese	Asia	Meng et al., 2021
7	1	USH2A	c.100C>T	p.R34*	Nonsense	E2	–	France	Europe	Maubaret et al., 2005
8	1	USH2A	c.100_101insT	p.R34Lfs*41	Frameshift	E2	–	Chinese	Asia	Jiang et al., 2015
9	1	USH2A	c.100_101insT	p.R34Lfs*41	Frameshift	E2	–	Chinese	Asia	Zhu et al., 2021
10	3	USH2A	c.187C>T	p.R63*	Nonsense	E2	–	European	Europe	Hartel et al., 2016
11	2	USH2A	c.236_239dup	p.Q81Yfs*28	Frameshift	E2	–	Iran	Asia	Adato et al., 2000
12	3	USH2A	c.236_239dup	p.Q81Yfs*28	Frameshift	E2	–	British	Europe	Leroy et al., 2001
13	1	USH2A	c.236_239dup	p.Q81Yfs*28	Frameshift	E2	–	Iraq	Asia	Auslender et al., 2008
14	1	USH2A	c.236_239dup	p.Q81Yfs*28	Frameshift	E2	–	European	Europe	Hartel et al., 2016
15	7	USH2A	c.236_239dup	p.Q81Yfs*28	Frameshift	E2	–	Israeli	Asia	Khalaileh et al., 2018
16	2	USH2A	c.236_239dup	p.Q81Yfs*28	Frameshift	E2	–	–	–	Bahena et al., 2022
17	1	USH2A	c.532dup	p.T178Nfs*4	Frameshift	E3	–	Lebanese	Asia	Reddy et al., 2014
18	7	USH2A	c.802G>A	p.G268R	Missense	E5	–	Israeli	Asia	Khalaileh et al., 2018
19	1	RP	c.802G>A	p.G268R	Missense	E5	–	Japanese	Asia	Koyanagi et al., 2019
20	1	USH2A	c.842C>A	p.T281K	Missense	E5	Lam NT	Turkish Cypriot	Asia	Le Quesne Stabej et al., 2012
21	1	USH2A	c.908G>A	p.R303H	Missense	E6	Lam NT	Italian	Europe	Eandi et al., 2017
22	1	USH2A	c.920_923dup	p.H308Qfs*16	Frameshift	E6	Lam NT	Danish	Europe	Dreyer et al., 2000
23	1	USH2A	c.920_923dup	p.H308Qfs*16	Frameshift	E6	Lam NT	European	Europe	Krawitz et al., 2014
24	1	USH2A	c.920_923dup	p.H308Qfs*16	Frameshift	E6	Lam NT	European	Europe	Baux et al., 2014
25	1	USH2A	c.920_923dup	p.H308Qfs*16	Frameshift	E6	Lam NT	European	Europe	Hartel et al., 2016
26	6	USH2A	c.1000C>T	p.R334W	Missense	E6	Lam NT	Morocco	Africa	Adato et al., 2000
27	2	USH2A	c.1000C>T	p.R334W	Missense	E6	Lam NT	Non-Ashkenazi Jews	–	Auslender et al., 2008
28	1	USH2A	c.1000C>T	p.R334W	Missense	E6	Lam NT	Chinese	Asia	Jiang et al., 2015
29	4	USH2A	c.1000C>T	p.R334W	Missense	E6	Lam NT	Israeli	Asia	Khalaileh et al., 2018
30	1	USH2A	c.1000C>T	p.R334W	Missense	E6	Lam NT	Chinese	Asia	Zhu et al., 2021

No.	Number of patients	Phenotype	Nucleotide change[a]	Amino acid change[a]	Variant type[b]	Exon/ Intron[c]	Domain	Geographical/ Ethnic distribution	Continent	Reference
31	3	USH2A	c.1036A>C	p.N346H	Missense	E6	Lam NT	Swedish	Europe	Sadeghi et al., 2013
32	4	USH2A	c.1036A>C	p.N346H	Missense	E6	Lam NT	European	Europe	Hartel et al., 2016
33	1	USH2A	c.1214del	p.N405Ifs*3	Frameshift	E7	Lam NT	Spanish	Europe	Garcia-Garcia et al., 2011
34	1	USH2A	–	p.W409*	Nonsense	E7	Lam NT	Dutch	Europe	Pennings et al., 2003
35	2	USH2A	–	p.W409*	Nonsense	E7	Lam NT	–	–	Pennings et al., 2004a
36	1	USH2A	c.1227G>A	p.W409*	Nonsense	E7	Lam NT	Dutch	Europe	Pennings et al., 2004b
37	3	USH2A	c.1227G>A	p.W409*	Nonsense	E7	Lam NT	European	Europe	Hartel et al., 2016
38	1	USH2A	c.1256G>T	p.C419F	Missense	E7	Lam NT	–	–	Pennings et al., 2004a
39	1	USH2A	c.1256G>T	p.C419F	Missense	E7	Lam NT	Caucasian	Europe	Le Quesne Stabej et al., 2012
40	2	USH2A	c.1256G>T	p.C419F	Missense	E7	Lam NT	European	Europe	Hartel et al., 2016
41	1	USH2A	c.1256G>T	p.C419F	Missense	E7	Lam NT	–	–	Hartel et al., 2017
42	1	RP	c.1397G>T	p.G466V	Missense	E8	Lam NT	Chinese	Asia	Dan et al., 2020
43	1	RP	c.1550G>C	p.R517T	Missense	E8	Lam NT	United States	America	Zampaglione et al., 2020
44	1	USH2A	c.1558T>C	p.C520R	Missense	E9	EGF Lam 1	European	Europe	Krawitz et al., 2014
45	1	USH2A	c.1558T>C	p.C520R	Missense	E9	EGF Lam 1	European	Europe	Bonnet et al., 2016
46	1	USH2A	c.1558del	p.C520Afs*71	Frameshift	E9	EGF Lam 1	United Kingdom	Europe	Molina-Ramírez et al., 2020
47	1	USH2A	c.1606T>C	p.C536R	Missense	E9	EGF Lam 1	Denmark	Europe	Dad et al., 2016
48	1	USH2A	c.1606T>C	p.C536R	Missense	E9	EGF Lam 1	German	Europe	Neuhaus et al., 2017
49	1[#]	USH2A	c.1663C>G	p.L555V	Missense	E10	EGF Lam 1	Spanish	Europe	Jaijo et al., 2010
50	1[#]	RP	c.1678C>G	p.P560A	Missense	E10	EGF Lam 1	Japanese	Asia	Koyanagi et al., 2020
51	1	USH2A	c.1859G>A	p.C620Y	Missense	E11	EGF Lam 2	European	Europe	Baux et al., 2014
52	1	USH2A	c.1876C>T	p.R626*	Nonsense	E11	EGF Lam 2	European	Europe	Ouyang et al., 2004

No.	Number of patients	Phenotype	Nucleotide change[a]	Amino acid change[a]	Variant type[b]	Exon/ Intron[c]	Domain	Geographical/ Ethnic distribution	Continent	Reference
53	1	USH2A	c.1876C>T	p.R626*	Nonsense	E11	EGF Lam 2	North America	America	Seyedahmadi et al., 2004
54	1	RP	c.1876C>T	p.R626*	Nonsense	E11	EGF Lam 2	–	–	Sandberg et al., 2008
55	1	USH2A	c.1876C>T	p.R626*	Nonsense	E11	EGF Lam 2	–	–	Lenassi et al., 2015
56	1	USH2A	c.1876C>T	p.R626*	Nonsense	E11	EGF Lam 2	European	Europe	Hartel et al., 2016
57	1	USH2A	c.1876C>T	p.R626*	Nonsense	E11	EGF Lam 2	European	Europe	Bonnet et al., 2016
58	1	USH2A	c.1876C>T	p.R626*	Nonsense	E11	EGF Lam 2	Chinese	Asia	Sun et al., 2018
59	1	RP	c.1876C>T	p.R626*	Nonsense	E11	EGF Lam 2	United States	America	Zampaglione et al., 2020
60	1	USH2A	c.1913G>T	p.C638F	Missense	E11	EGF Lam 2	European	Europe	Baux et al., 2014
61	1	USH2A	c.2017T>A	p.C673S	Missense	E12	EGF Lam 3	Chinese	Asia	Dan et al., 2020
62	1	USH2A	c.2209C>T	p.R737*	Nonsense	E13	EGF Lam 4	Israeli	Asia	Kaiserman et al., 2007
63	3	USH2A	c.2209C>T	p.R737*	Nonsense	E13	EGF Lam 4	Iraq	Asia	Auslender et al., 2008
64	1	USH2A	c.2209C>T	p.R737*	Nonsense	E13	EGF Lam 4	Israeli	Asia	Khalaileh et al., 2018
65	1	RP	c.2276G>T	p.C759F	Missense	E13	EGF Lam 5	–	–	Rivolta et al., 2002
66	2	RP	c.2276G>T	p.C759F	Missense	E13	EGF Lam 5	Spanish	Europe	Bernal et al., 2003
67	1	RP	c.2276G>T	p.C759F	Missense	E13	EGF Lam 5	Spanish	Europe	Aller et al., 2004
68	2	RP	c.2276G>T	p.C759F	Missense	E13	EGF Lam 5	North America	America	Seyedahmadi et al., 2004
69	3	RP	c.2276G>T	p.C759F	Missense	E13	EGF Lam 5	–	–	Sandberg et al., 2008
70	6	RP	c.2276G>T	p.C759F	Missense	E13	EGF Lam 5	Spanish	Europe	Ávila-Fernández et al., 2010

No.	Number of patients	Phenotype	Nucleotide change[a]	Amino acid change[a]	Variant type[b]	Exon/ Intron[c]	Domain	Geographical/ Ethnic distribution	Continent	Reference
71	1	RP	c.2276G>T	p.C759F	Missense	E13	EGF Lam 5	European	Europe	Glöckle et al., 2014
72	1	USH2A	c.2276G>T	p.C759F	Missense	E13	EGF Lam 5	European	Europe	Baux et al., 2014
73	1	RP	c.2276G>T	p.C759F	Missense	E13	EGF Lam 5	European	Europe	Lenassi et al., 2015
74	2	RP	c.2276G>T	p.C759F	Missense	E13	EGF Lam 5	–	–	Lenassi et al., 2015
75	1	RP	c.2276G>T	p.C759F	Missense	E13	EGF Lam 5	–	–	Sengillo et al., 2017
76	6	RP	c.2276G>T	p.C759F	Missense	E13	EGF Lam 5	non-Asian	–	DuPont et al., 2018
77	14	RP	c.2276G>T	p.C759F	Missense	E13	EGF Lam 5	Spanish	Europe	Pérez-Carro et al., 2018
78	1	USH2A	c.2276G>T	p.C759F	Missense	E13	EGF Lam 5	United Kingdom	Europe	Molina-Ramírez et al., 2020
79	1	RP	c.2276G>T	p.C759F	Missense	E13	EGF Lam 5	Spanish	Europe	García Bohórquez et al., 2021
80	1	RP	c.2296T>C	p.C766R	Missense	E13	EGF Lam 5	European	Europe	Karali et al., 2019
81	7	USH2A	c.2299del	p.E767Sfs*21	Frameshift	E13	EGF Lam 5	European	Europe	Eudy et al., 1998
82	1	USH2A	c.2299del	p.E767Sfs*21	Frameshift	E13	EGF Lam 5	African	Africa	Eudy et al., 1998
83	4	USH2A	c.2299del	p.E767Sfs*21	Frameshift	E13	EGF Lam 5	Danish	Europe	Dreyer et al., 2000
84	2	USH2A	c.2299del	p.E767Sfs*21	Frameshift	E13	EGF Lam 5	Norwegian	Europe	Dreyer et al., 2000
85	4	USH2A	c.2299del	p.E767Sfs*21	Frameshift	E13	EGF Lam 5	Spanish	Europe	Nájera et al., 2002
86	5	USH2A	c.2299del	p.E767Sfs*21	Frameshift	E13	EGF Lam 5	European	Europe	Ouyang et al., 2004
87	6	USH2A	c.2299del	p.E767Sfs*21	Frameshift	E13	EGF Lam 5	–	–	Pennings et al., 2004a
88	5	USH2A	c.2299del	p.E767Sfs*21	Frameshift	E13	EGF Lam 5	Dutch	Europe	Pennings et al., 2004b

No.	Number of patients	Phenotype	Nucleotide change[a]	Amino acid change[a]	Variant type[b]	Exon/ Intron[c]	Domain	Geographical/ Ethnic distribution	Continent	Reference
89	1	USH2A	c.2299del	p.E767Sfs*21	Frameshift	E13	EGF Lam 5	North America	America	Seyedahmadi et al., 2004
90	1	USH2A	c.2299del	p.E767Sfs*21	Frameshift	E13	EGF Lam 5	France	Europe	Maubaret et al., 2005
91	2	USH2A	c.2299del	p.E767Sfs*21	Frameshift	E13	EGF Lam 5	Spanish	Europe	Bernal et al., 2005
92	1	USH2A	c.2299del	p.E767Sfs*21	Frameshift	E13	EGF Lam 5	Caucasian	Europe	Baux et al., 2007
93	1	RP	c.2299del	p.E767Sfs*21	Frameshift	E13	EGF Lam 5	–	–	Sandberg et al., 2008
94	1	USH2A	c.2299del	p.E767Sfs*21	Frameshift	E13	EGF Lam 5	Dutch	Europe	Leijendeckers et al., 2009
95	2	USH2A	c.2299del	p.E767Sfs*21	Frameshift	E13	EGF Lam 5	American	America	Yan et al., 2009
96	2	USH2A	c.2299del	p.E767Sfs*21	Frameshift	E13	EGF Lam 5	Spanish	Europe	Jaijo et al., 2010
97	1	USH2A	c.2299del	p.E767Sfs*21	Frameshift	E13	EGF Lam 5	Caucasian	Europe	Le Quesne Stabej et al., 2012
98	2	USH2A	c.2299del	p.E767Sfs*21	Frameshift	E13	EGF Lam 5	Australia	Oceania	Sadeghi et al., 2013
99	5	USH2A	c.2299del	p.E767Sfs*21	Frameshift	E13	EGF Lam 5	United Kingdom	Europe	Lenassi et al., 2014
100	1	USH2A	c.2299del	p.E767Sfs*21	Frameshift	E13	EGF Lam 5	European	Europe	Krawitz et al., 2014
101	4	USH2A	c.2299del	p.E767Sfs*21	Frameshift	E13	EGF Lam 5	European	Europe	Baux et al., 2014
102	11	USH2A	c.2299del	p.E767Sfs*21	Frameshift	E13	EGF Lam 5	–	–	Blanco-Kelly et al., 2015
103	3	USH2A	c.2299del	p.E767Sfs*21	Frameshift	E13	EGF Lam 5	Denmark	Europe	Dad et al., 2016
104	1	USH2A	c.2299del	p.E767Sfs*21	Frameshift	E13	EGF Lam 5	Algerian	Africa	Abdi et al., 2016
105	15	USH2A	c.2299del	p.E767Sfs*21	Frameshift	E13	EGF Lam 5	European	Europe	Hartel et al., 2016
106	6	USH2A	c.2299del	p.E767Sfs*21	Frameshift	E13	EGF Lam 5	European	Europe	Bonnet et al., 2016

No.	Number of patients	Phenotype	Nucleotide change[a]	Amino acid change[a]	Variant type[b]	Exon/ Intron[c]	Domain	Geographical/ Ethnic distribution	Continent	Reference
107	1	USH2A	c.2299del	p.E767Sfs*21	Frameshift	E13	EGF Lam 5	–	–	Hartel et al., 2017
108	2	USH2A	c.2299del	p.E767Sfs*21	Frameshift	E13	EGF Lam 5	German	Europe	Neuhaus et al., 2017
109	1	USH2A	c.2299del	p.E767Sfs*21	Frameshift	E13	EGF Lam 5	–	–	Sengillo et al., 2017
110	2	USH2A	c.2299del	p.E767Sfs*21	Frameshift	E13	EGF Lam 5	Spanish	Europe	Fuster-García et al., 2018
111	1	USH2A	c.2299del	p.E767Sfs*21	Frameshift	E13	EGF Lam 5	Cuban	America	Santana et al., 2019
112	1	RP	c.2299del	p.E767Sfs*21	Frameshift	E13	EGF Lam 5	United States	America	Zampaglione et al., 2020
113	3	USH2A	c.2299del	p.E767Sfs*21	Frameshift	E13	EGF Lam 5	–	–	Toms et al., 2020
114	1	USH2A	c.2299del	p.E767Sfs*21	Frameshift	E13	EGF Lam 5	Italy	Europe	Falsini et al., 2021
115	3	USH2A	c.2299del	p.E767Sfs*21	Frameshift	E13	EGF Lam 5	–	–	Wafa et al., 2021
116	1	RP	c.2332G>T	p.D778Y	Missense	E13	EGF Lam 5	African	Africa	Lenassi et al., 2015
117	1	USH2A	c.2610C>A	p.C870*	Nonsense	E13	EGF Lam 7	Turkish Cypriot	Asia	Le Quesne Stabej et al., 2012
118	2	USH2A	c.2610C>A	p.C870*	Nonsense	E13	EGF Lam 7	European	Europe	Bonnet et al., 2016
119	1	USH2A	c.2610C>A	p.C870*	Nonsense	E13	EGF Lam 7	–	–	Toms et al., 2020
120	1	USH2A	c.2802T>G	p.C934W	Missense	E13	EGF Lam 8	Chinese	Asia	Jiang et al., 2015
121	8	RP	c.2802T>G	p.C934W	Missense	E13	EGF Lam 8	Japanese	Asia	Koyanagi et al., 2019
122	1	USH2A	c.2802T>G	p.C934W	Missense	E13	EGF Lam 8	–	–	Toms et al., 2020
123	3	RP	c.2802T>G	p.C934W	Missense	E13	EGF Lam 8	Chinese	Asia	Gao et al., 2021
124	5	RP	c.2802T>G	p.C934W	Missense	E13	EGF Lam 8	Chinese	Asia	Zhu et al., 2021

No.	Number of patients	Phenotype	Nucleotide change[a]	Amino acid change[a]	Variant type[b]	Exon/ Intron[c]	Domain	Geographical/ Ethnic distribution	Continent	Reference
125	1	USH2A	c.2802T>G	p.C934W	Missense	E13	EGF Lam 8	Chinese	Asia	Zhu et al., 2021
126	1	RP	c.2802T>G	p.C934W	Missense	E13	EGF Lam 8	Chinese	Asia	Meng et al., 2021
127	1	USH2A	c.2878_2879del	p.N960Sfs*4	Frameshift	E14	EGF Lam 9	Danish	Europe	Dreyer et al., 2000
128	1	USH2A	c.2898del	p.T967Lfs*44	Frameshift	E14	EGF Lam 9	Spanish	Europe	Jaijo et al., 2010
129	1	USH2A	c.2950C>T	p.Q984*	Nonsense	E14	EGF Lam 9	Spanish	Europe	Fuster-García et al., 2018
130	2	USH2A	c.2983C>T	p.Q995*	Nonsense	E14	EGF Lam 9	European	Europe	Hartel et al., 2016
131	1	RP	c.3724C>T	p.P1242S	Missense	E17	FN III 2	South Asian	Asia	Lenassi et al., 2015
132	1	USH2A	c.3737dup	p.S1247Kfs*4	Frameshift	E17	FN III 3	European	Europe	Baux et al., 2014
133	1	USH2A	c.4073del	p.G1358Efs*8	Frameshift	E18	FN III 3	Chinese	Asia	Sun et al., 2018
134	1	RP	c.4222C>T	p.Q1408*	Nonsense	E19	FN III 4	United Kingdom	Europe	Molina-Ramírez et al., 2020
135	1	USH2A	c.4314del	p.I1439Yfs*15	Frameshift	E20	FN III 4	Asian	Asia	Neuhaus et al., 2017
136	1	USH2A	c.4338_4339del	p.C1447Qfs*29	Frameshift	E20	FN III 4	North America	America	Seyedahmadi et al., 2004
137	1	RP	c.4338_4339del	p.C1447Qfs*29	Frameshift	E20	FN III 4	–	–	Sandberg et al., 2008
138	4	USH2A	c.4338_4339del	p.C1447Qfs*29	Frameshift	E20	FN III 4	Canadian	America	Ebermann et al., 2009
139	2	USH2A	c.4338_4339del	p.C1447Qfs*29	Frameshift	E20	FN III 4	French-Canadian	America	Ebermann et al., 2010
140	1	USH2A	c.4338_4339del	p.C1447Qfs*29	Frameshift	E20	FN III 4	–	–	Wafa et al., 2021
141	1	USH2A	c.4354dup	p.C1452Lfs*25	Frameshift	E20	FN III 4	Indian	Asia	Le Quesne Stabej et al., 2012
142	1	USH2A	c.4382del	p.T1462Lfs*2	Frameshift	E20	FN III 4	Chinese	Asia	Shu et al., 2015
143	1	USH2A	c.4385C>T	p.T1462I	Missense	E20	FN III 4	Spanish	Europe	Fuster-García et al., 2018
144	2	USH2A	c.4474G>T	p.E1492*	Nonsense	E21	–	Spanish	Europe	Garcia-Garcia et al., 2011
145	1	RP	c.4616C>T	p.T1539I	Missense	E21	Lam G 1	Chinese	Asia	Gao et al., 2021
146	1	USH2A	c.4645C>T	p.R1549*	Nonsense	E22	Lam G 1	European	Europe	Baux et al., 2014
147	1	USH2A	c.4732C>T	p.R1578C	Missense	E22	Lam G 1	–	–	Bahena et al., 2022
148	1	USH2A	c.5018T>C	p.L1673P	Missense	E25	Lam G 1	European	Europe	Hartel et al., 2016

No.	Number of patients	Phenotype	Nucleotide change[a]	Amino acid change[a]	Variant type[b]	Exon/ Intron[c]	Domain	Geographical/ Ethnic distribution	Continent	Reference
149	1	USH2A	c.5018T>C	p.L1673P	Missense	E25	Lam G 1	–	–	Hartel et al., 2017
150	1	USH2A	c.5189_5199del	p.Y1730Wfs*6	Frameshift	E26	Lam G 2	Italian	Europe	Lenarduzzi et al., 2015
151	1	RP	c.5200G>C	p.G1734R	Missense	E26	Lam G 2	Chinese	Asia	Liu et al., 2010
152	1	USH2A	c.5221T>C	p.S1741P	Missense	E26	Lam G 2	Italy	Europe	Falsini et al., 2021
153	1	USH2A	c.5418_5424del	p.K1807Afs*8	Frameshift	E27	Lam G 2	Italian	Europe	Lenarduzzi et al., 2015
154	1	USH2A	c.5519G>T	p.G1840V	Missense	E27	Lam G 2	Israeli	Asia	Khalaileh et al., 2018
155	1[#]	USH2A	c.5698T>G	p.C1900G	Missense	E28	FN III 5	European	Europe	Bonnet et al., 2016
156	1[#]	USH2A	c.5933_5940del	p.P1978Qfs*5	Frameshift	E30	FN III 6	Italian	Europe	Eandi et al., 2017
157	1[#]	USH2A	c.5950_5960dup	p.Y1987*	Nonsense	E30	FN III 6	Italian	Europe	Eandi et al., 2017
158	1	USH2A	c.6741del	p.A2249Pfs*30	Frameshift	E35	FN III 9	Chinese	Asia	Sun et al., 2018
159	1	USH2A	c.6862G>T	p.E2288*	Nonsense	E36	FN III 9	American	America	Yan et al., 2009
160	1	USH2A	c.6967C>T	p.R2323*	Nonsense	E37	FN III 9	Chinese	Asia	Sun et al., 2018
161	1	RP	c.7068T>G	p.N2356K	Missense	E37	FN III 10	Chinese	Asia	Meng et al., 2021
162	1	USH2A	c.7198del	p.D2400Mfs*13	Frameshift	E38	FN III 10	German	Europe	Neuhaus et al., 2017
163	3	USH2A	c.7334C>T	p.S2445F	Missense	E39	FN III 11	Pakistani	Asia	Ahmed et al., 2021
164	1	USH2A	c.7789A>T	p.K2597*	Nonsense	E41	FN III 12	–	–	Toms et al., 2020
165	1	USH2A	c.7915T>C	p.S2639P	Missense	E41	FN III 13	European	Europe	Bonnet et al., 2016
166	1	USH2A	c.7932G>A	p.W2644*	Nonsense	E41	FN III 13	Spanish	Europe	Fuster-García et al., 2018
167	1	USH2A	c.8079G>A	p.W2693*	Nonsense	E41	FN III 13	European	Europe	Hartel et al., 2016
168	1	USH2A	c.8079G>A	p.W2693*	Nonsense	E41	FN III 13	–	–	Hartel et al., 2017
169	1	RP	c.8167C>T	p.R2723*	Nonsense	E41	–	–	–	Sandberg et al., 2008
170	1	USH2A	c.8167C>T	p.R2723*	Nonsense	E41	–	United States	America	McGee et al., 2010
171	2	USH2A	c.8232G>C	p.W2744C	Missense	E42	FN III 14	Chinese	Asia	Gao et al., 2021
172	1	RP	c.8254G>A	p.G2752R	Missense	E42	FN III 14	Japanese	Asia	Koyanagi et al., 2019
173	1[#]	RP	–	p.G2752R	Missense	E42	FN III 14	Japanese	Asia	Koyanagi et al., 2020
174	1	RP	c.8254G>A	p.G2752R	Missense	E42	FN III 14	Japanese	Asia	Inaba et al., 2020
175	1	USH2A	c.8396del	p.G2799Vfs*31	Frameshift	E42	FN III 14	Japanese	Asia	Inaba et al., 2020
176	1	USH2A	c.8483_8486del	p.S2828*	Nonsense	E42	FN III 15	Chinese	Asia	Xing et al., 2020
177	1	USH2A	c.8497dup	p.S2833Kfs*2	Frameshift	E42	FN III 15	–	–	Bahena et al., 2022
178	4	USH2A	c.8681G>A	p.R2894K	Missense	E43	FN III 15	Jordanian	Asia	Reddy et al., 2014
179	1	USH2A	c.8906C>G	p.S2969*	Nonsense	E45	FN III 16	Italian	Europe	Sodi et al., 2014
180	1	USH2A	c.8906C>G	p.S2969*	Nonsense	E45	FN III 16	European	Europe	Bonnet et al., 2016

No.	Number of patients	Phenotype	Nucleotide change[a]	Amino acid change[a]	Variant type[b]	Exon/ Intron[c]	Domain	Geographical/ Ethnic distribution	Continent	Reference
181	1	USH2A	c.8917_8198del	p.L2973Kfs*79	Frameshift	E45	FN III 16	–	–	Wafa et al., 2021
182	1	USH2A	c.9120G>A	p.W3040*	Nonsense	E46	FN III 17	Chinese	Asia	Zhu et al., 2021
183	1	USH2A	c.9345_9346del	p.P3116Hfs*13	Frameshift	E47	FN III 18	European	Europe	Baux et al., 2014
184	1	USH2A	c.9345_9346del	p.P3116Hfs*13	Frameshift	E47	FN III 18	European	Europe	Bonnet et al., 2016
185	1	USH2A	c.9424G>T	p.G3142*	Nonsense	E48	FN III 18	German	Europe	Neuhaus et al., 2017
186	1	USH2A	c.9433C>T	p.L3145F	Missense	E48	FN III 18	Spanish	Europe	Perez-Carro et al., 2016
187	2	USH2A	c.9469C>T	p.Q3157*	Nonsense	E48	FN III 18	Chinese	Asia	Zhu et al., 2021
188	1	USH2A	c.9723C>A	p.Y3241*	Nonsense	E49	–	Chinese	Asia	Jiang et al., 2015
189	1	USH2A	c.9723C>A	p.Y3241*	Nonsense	E49	–	Chinese	Asia	Zhu et al., 2021
190	1	USH2A	c.9799T>C	p.C3267R	Missense	E50	–	Spanish	Europe	Jaijo et al., 2010
191	1	USH2A	c.9799T>C	p.C3267R	Missense	E50	–	Spanish	Europe	Fuster-García et al., 2018
192	1	USH2A	c.9815C>T	p.P3272L	Missense	E50	–	Italy	Europe	Falsini et al., 2021
193	2	RP	c.9815C>T	p.P3272L	Missense	E50	–	Italy	Europe	Falsini et al., 2021
194	1	USH2A	c.9976C>T	p.Q3326*	Nonsense	E51	–	–	–	Toms et al., 2020
195	1	RP	c.10073G>A	p.C3358Y	Missense	E51	–	–	–	Sengillo et al., 2017
196	1	RP	c.10342G>A	p.E3448K	Missense	E52	FN III 19	–	–	Comander et al., 2017
197	1	RP	c.10342G>A	p.E3448K	Missense	E52	FN III 19	United Kingdom	Europe	Molina-Ramírez et al., 2020
198	1	USH2A	c.10561T>C	p.W3521R	Missense	E53	FN III 20	European	Europe	Hartel et al., 2016
199	1	USH2A	c.10607T>G	p.L3536R	Missense	E54	FN III 20	European	Europe	Baux et al., 2014
200	1	USH2A	c.10636G>A	p.G3546R	Missense	E54	FN III 20	Spanish	Europe	Garcia-Garcia et al., 2011
201	1	USH2A	c.10636G>A	p.G3546R	Missense	E54	FN III 20	Spanish	Europe	de Castro-Miró et al., 2014
202	1	USH2A	c.10636G>A	p.G3546R	Missense	E54	FN III 20	European	Europe	Baux et al., 2014
203	1	USH2A	c.10699del	p.L3567*	Nonsense	E54	FN III 20	Italy	Europe	Falsini et al., 2021
204	1	USH2A	c.10712C>T	p.T3571M	Missense	E54	FN III 20	Spanish	Europe	Jaijo et al., 2010
205	1	USH2A	c.10712C>T	p.T3571M	Missense	E54	FN III 20	Italian	Europe	Lenarduzzi et al., 2015
206	2	USH2A	c.10712C>T	p.T3571M	Missense	E54	FN III 20	European	Europe	Bonnet et al., 2016
207	1	USH2A	c.10712C>T	p.T3571M	Missense	E54	FN III 20	–	–	Sengillo et al., 2017
208	1	USH2A	c.10712C>T	p.T3571M	Missense	E54	FN III 20	Spanish	Europe	Fuster-García et al., 2018

No.	Number of patients	Phenotype	Nucleotide change[a]	Amino acid change[a]	Variant type[b]	Exon/ Intron[c]	Domain	Geographical/ Ethnic distribution	Continent	Reference
209	1	RP	c.10721G>T	p.G3574V	Missense	E54	FN III 20	Turkey	Asia	Coppieters et al., 2014
210	1	RP	c.10931C>T	p.T3644M	Missense	E55	FN III 21	Japanese	Asia	Katagiri et al., 2014
211	1	RP	c.10999A>C	p.T3667P	Missense	E56	FN III 21	Japanese	Asia	Koyanagi et al., 2019
212	1	USH2A	c.11095G>T	p.E3699*	Nonsense	E57	FN III 22	European	Europe	Baux et al., 2014
213	1	USH2A	c.11105G>A	p.W3702*	Nonsense	E57	FN III 22	European	Europe	Krawitz et al., 2014
214	2	USH2A	c.11105G>A	p.W3702*	Nonsense	E57	FN III 22	European	Europe	Bonnet et al., 2016
215	1	RP	c.11156G>A	p.R3719H	Missense	E57	FN III 22	Japanese	Asia	Koyanagi et al., 2019
216	1	USH2A	c.11156G>A	p.R3719H	Missense	E57	FN III 22	Chinese	Asia	Dan et al., 2020
217	1	RP	c.11156G>A	p.R3719H	Missense	E57	FN III 22	Chinese	Asia	Gao et al., 2021
218	1	RP	c.11156G>A	p.R3719H	Missense	E57	FN III 22	Chinese	Asia	Zhu et al., 2021
219	1	USH2A	c.11194C>T	p.Q3732*	Nonsense	E57	FN III 22	Italian	Europe	Sodi et al., 2014
220	1	RP	c.11235C>G	p.Y3745*	Nonsense	E58	FN III 22	Chinese	Asia	Chen et al., 2014
221	1	USH2A	c.11284dup	p.D3762Gfs*19	Frameshift	E58	FN III 22	European	Europe	Baux et al., 2014
222	1	USH2A	c.11357del	p.P3786Lfs*6	Frameshift	E58	FN III 23	–	–	Bahena et al., 2022
223	1	RP	c.11387C>T	p.P3796L	Missense	E58	FN III 23	Mexican	America	Zenteno et al., 2020
224	2	USH2A	c.11404G>T	p.E3802*	Nonsense	E59	FN III 23	Spanish	Europe	Fuster-García et al., 2018
225	1	RP	c.11533C>T	p.Q3845*	Nonsense	E59	FN III 23	–	–	Sandberg et al., 2008
226	1	USH2A	c.11533C>T	p.Q3845*	Nonsense	E59	FN III 23	United States	America	McGee et al., 2010
227	1	USH2A	c.11700C>A	p.Y3900*	Nonsense	E60	FN III 24	–	–	Toms et al., 2020
228	1	USH2A	c.11806A>C	p.T3936P	Missense	E61	FN III 24	Chinese	Asia	Meng et al., 2021
229	1	USH2A	c.11864G>A	p.W3955*	Nonsense	E61	FN III 24	Italian	Europe	Lenarduzzi et al., 2015
230	3	USH2A	c.11864G>A	p.W3955*	Nonsense	E61	FN III 24	European	Europe	Hartel et al., 2016
231	11	USH2A	c.11864G>A	p.W3955*	Nonsense	E61	FN III 24	European	Europe	Bonnet et al., 2016
232	1	USH2A	c.11864G>A	p.W3955*	Nonsense	E61	FN III 24	Turkey	Asia	Neuhaus et al., 2017
233	1	USH2A	c.11864G>A	p.W3955*	Nonsense	E61	FN III 24	Russian	Europe	Neuhaus et al., 2017
234	10	USH2A	c.11864G>A	p.W3955*	Nonsense	E61	FN III 24	Slovenian	Europe	Zupan et al., 2019
235	1	USH2A	c.11864G>A	p.W3955*	Nonsense	E61	FN III 24	–	–	Wafa et al., 2021
236	3	USH2A	c.11907del	p.A3970Lfs*14	Frameshift	E61	FN III 25	Lebanese	Asia	Reddy et al., 2014
237	1	USH2A	c.11955G>C	p.W3985C	Missense	E61	FN III 25	–	–	Bahena et al., 2022
238	1	USH2A	c.12093del	p.Y4031*	Nonsense	E62	FN III 25	Spanish	Europe	Fuster-García et al., 2018
239	1	USH2A	c.12104C>T	p.P4035L	Missense	E62	FN III 25	Chinese	Asia	Zhu et al., 2021

No.	Number of patients	Phenotype	Nucleotide change[a]	Amino acid change[a]	Variant type[b]	Exon/ Intron[c]	Domain	Geographical/ Ethnic distribution	Continent	Reference
240	1	USH2A	c.12172_12174delinsTAAA	p.L4058*	Nonsense	E62	FN III 25	European	Europe	Baux et al., 2014
241	1	USH2A	c.12234_12235del	p.N4079Wfs*19	Frameshift	E62	FN III 26	European	Europe	Bonnet et al., 2016
242	1	USH2A	c.12275_12279delinsTGTGATGTGATTAAGGT	p.R4092_L5202delinsM	Small indel	E62	FN III 26	Chinese	Asia	Sun et al., 2018
243	1[#]	USH2A	c.12343C>T	p.R4115C	Missense	E63	FN III 26	European	Europe	Bonnet et al., 2016
244	1	USH2A	c.12394del	p.L4132Wfs*35	Frameshift	E63	FN III 26	–	–	Bahena et al., 2022
245	1	RP	c.12574C>T	p.R4192C	Missense	E63	FN III 27	Belgium	Europe	Coppieters et al., 2014
246	1	RP	c.12575G>A	p.R4192H	Missense	E63	FN III 27	Spanish	Europe	García Bohórquez et al., 2021
247	1	USH2A	c.12700A>C	p.T4234P	Missense	E63	FN III 27	Italian	Europe	Lenarduzzi et al., 2015
248	1	USH2A	c.12708T>A	p.C4236*	Nonsense	E63	FN III 27	Japanese	Asia	Nakanishi et al., 2011
249	1	USH2A	c.12806C>G	p.P4269R	Missense	E63	FN III 28	United States	America	McGee et al., 2010
250	1	USH2A	c.12845T>C	p.L4282P	Missense	E63	FN III 28	European	Europe	Bonnet et al., 2016
251	2	RP	c.12874A>G	p.N4292D	Missense	E63	FN III 28	Asian	Asia	Watson et al., 2014
252	1	USH2A	c.13010C>T	p.T4337M	Missense	E63	FN III 28	Spanish	Europe	Aller et al., 2006
253	1	USH2A	c.13022G>T	p.C4341F	Missense	E63	FN III 28	–	–	Wafa et al., 2021
254	1[#]	USH2A	c.13274C>T	p.T4425M	Missense	E63	FN III 29	European	Europe	Bonnet et al., 2016
255	1	RP	c.13335_13347delinsCTTG	p.E4445_S4449delinsDL	Small indel	E63	FN III 29	European	Europe	Glöckle et al., 2014
256	1	RP	c.13422C>G	p.I4474M	Missense	E63	FN III 30	United States	America	Zampaglione et al., 2020
257	1	RP	c.13465G>A	p.G4489S	Missense	E63	FN III 30	Chinese	Asia	Gao et al., 2021
258	1	RP	c.13466G>A	p.G4489D	Missense	E63	FN III 30	Japanese	Asia	Oishi et al., 2014
259	1	RP	c.13491_13499dup	p.T4498_T4500dup	Small indel	E63	FN III 30	–	–	Sengillo et al., 2017
260	2	RP	c.13514A>G	p.Y4505C	Missense	E63	FN III 30	United States	America	Zampaglione et al., 2020
261	1	USH2A	c.13576C>T	p.R4526*	Nonsense	E63	FN III 30	Japanese	Asia	Nakanishi et al., 2011
262	1[#]	USH2A	c.13576C>T	p.R4526*	Nonsense	E63	FN III 30	Japanese	Asia	Inaba et al., 2020
263	1[#]	USH2A	c.13847G>T	p.G4616V	Missense	E64	FN III 31	Japanese	Asia	Inaba et al., 2020
264	1	USH2A	c.14023A>T	p.R4675*	Nonsense	E64	FN III 32	Israeli	Asia	Khalaileh et al., 2018
265	2	USH2A	c.14031dup	p.A4678Sfs*5	Frameshift	E64	FN III 32	Lebanese	Asia	Reddy et al., 2014

No.	Number of patients	Phenotype	Nucleotide change[a]	Amino acid change[a]	Variant type[b]	Exon/ Intron[c]	Domain	Geographical/ Ethnic distribution	Continent	Reference
266	1	USH2A	c.14131C>T	p.Q4711*	Nonsense	E64	FN III 32	European	Europe	Krawitz et al., 2014
267	1	RP	c.14219C>A	p.A4740D	Missense	E65	FN III 33	United States	America	Zampaglione et al., 2020
268	1	USH2A	c.14225_14232dup	p.V4745Rfs*4	Frameshift	E65	FN III 33	European	Europe	Baux et al., 2014
269	1	RP	c.14243C>T	p.S4748F	Missense	E65	FN III 33	Japanese	Asia	Oishi et al., 2014
270	1	RP	c.14243C>T	p.S4748F	Missense	E65	FN III 33	Japanese	Asia	Inaba et al., 2020
271	1	USH2A	c.14248C>T	p.Q4750*	Nonsense	E65	FN III 33	Italy	Europe	Falsini et al., 2021
272	1	RP	c.14285A>G	p.N4762S	Missense	E65	FN III 33	Chinese	Asia	Sun et al., 2020
273	3	RP	c.14287G>C	p.G4763R	Missense	E65	FN III 33	Chinese	Asia	Chen et al., 2014
274	3	USH2A	c.14424C>A	p.C4808*	Nonsense	E66	FN III 33	Israeli	Asia	Khalaileh et al., 2018
275	1	USH2A	c.14439_14454del	p.C4813*	Nonsense	E66	FN III 33	Turkey	Asia	Neuhaus et al., 2017
276	1[#]	USH2A	c.14519T>C	p.L4840P	Missense	E66	FN III 34	European	Europe	Bonnet et al., 2016
277	1	USH2A	c.14586T>G	p.Y4862*	Nonsense	E67	FN III 34	European	Europe	Baux et al., 2014
278	1	USH2A	c.14803C>T	p.R4935*	Nonsense	E68	–	European	Europe	Baux et al., 2014
279	3	RP	c.14926G>A	p.G4976S	Missense	E68	–	Iranian	Asia	Salmaninejad et al., 2020
280	2	USH2A	c.14977_14978del	p.F4993Pfs*7	Frameshift	E69	–	European	Europe	Bonnet et al., 2016
281	1	USH2A	c.15017C>T	p.T5006M	Missense	E69	–	Algerian	Africa	Abdi et al., 2016
282	1	USH2A	c.15017C>T	p.T5006M	Missense	E69	–	Kingdom of Saudi Arabia	Asia	Neuhaus et al., 2017
283	1	RP	c.15178T>C	p.S5060P	Missense	E70	TM	Chinese	Asia	Zhu et al., 2021
284	1	RP	c.15233C>G	p.P5078R	Missense	E70	–	Japanese	Asia	Oishi et al., 2014
285	1	USH2A	c.15380del	p.P5127Rfs*8	Frameshift	E71	–	European	Europe	Bonnet et al., 2016
286	1	USH2A	c.15575_15579del	p.K5192Tfs*48	Frameshift	E72	–	Chinese	Asia	Gao et al., 2021
287	1	USH2A	c.785-6636_1840+208del (Del exon 5-10)	–	Gross deletion	E5-10	–	European	Europe	Bonnet et al., 2016
288	2	USH2A	c.1551-?_2993+?del (Del exon 9-14）	–	Gross deletion	E9-14	EGF Lam 1	Spanish	Europe	Bernal et al., 2005
289	1	RP	Del exon 14	–	Gross deletion	E14	EGF Lam 8	European	Europe	Glöckle et al., 2014
290	1	USH2A	Del exon 14	–	Gross deletion	E14	EGF Lam 8	European	Europe	Glöckle et al., 2014
291	1	USH2A	Del exon 14	–	Gross deletion	E14	EGF Lam 8	Syria	Asia	Neuhaus et al., 2017

No.	Number of patients	Phenotype	Nucleotide change[a]	Amino acid change[a]	Variant type[b]	Exon/ Intron[c]	Domain	Geographical/ Ethnic distribution	Continent	Reference
292	1	USH2A	c.4286_4396+16del (Del exon 20-intron 20)	–	Gross deletion	E20-I20	FN III 4	European	Europe	Baux et al., 2014
293	1	USH2A	c.4627+25435_4987+660del (Del exon 22-24)	–	Gross deletion	E22-24	Lam G 1	European	Europe	Bonnet et al., 2016
294	1	USH2A	c.4628-30487_6325+8822del (Del exon 22-32)	–	Gross deletion	E22-32	Lam G 1	European	Europe	Hartel et al., 2016
295	1	USH2A	Del exon 23-32	–	Gross deletion	E23-32	Lam G 1	Italian	Europe	Eandi et al., 2017
296	2	USH2A	c.7121-8313_11048-962delinsN[12] (Del exon 38-56)	–	Complex rearrangement	E38-56	FN III 10	European	Europe	Hartel et al., 2016
297	1	USH2A	Del exon 45-47	–	Gross deletion	E45-47	FN III 16	Syria	Asia	Neuhaus et al., 2017
298	1	USH2A	Del exon 47	–	Gross deletion	E47	FN III 17	Greek	Europe	Le Quesne Stabej et al., 2012
299	1	USH2A	c.9372-?_9570+?del (Del exon 48)	–	Gross deletion	E48	FN III 18	European	Europe	Hartel et al., 2016
300	1	USH2A	Del exon 48	–	Gross deletion	E48	FN III 18	Turkey	Asia	Neuhaus et al., 2017
301	1	USH2A	Del exon 50-55	–	Gross deletion	E50-55	–	Kashmiri	Asia	Le Quesne Stabej et al., 2012
302	2	USH2A	c.486-1G>C	–	Splicing	I2	–	Kingdom of Saudi Arabia	Asia	Neuhaus et al., 2017
303	1[#]	USH2A	c.1841-2A>G	–	Splicing	I10	–	Spanish	Europe	Jaijo et al., 2010
304	1	USH2A	c.1841-2A>G	–	Splicing	I10	–	Caucasian	Europe	Le Quesne Stabej et al., 2012
305	1	USH2A	c.1841-2A>G	–	Splicing	I10	–	European	Europe	Baux et al., 2014
306	1	USH2A	c.1841-2A>G	–	Splicing	I10	–	Italian	Europe	Sodi et al., 2014
307	1	USH2A	c.1841-2A>G	–	Splicing	I10	–	Cuban	America	Santana et al., 2019
308	1	USH2A	c.1841-2A>G	–	Splicing	I10	–	Mexican	America	Zenteno et al., 2020
309	1	RP	c.2167+5G>A	–	Splicing	I12	–	Spanish	Europe	Ávila-Fernández et al., 2010
310	1	USH2A	c.2809+1G>A	–	Splicing	I13	–	European	Europe	Baux et al., 2014
311	1	RP	c.4758+3A>G	–	Splicing	I22	–	Chinese	Asia	Meng et al., 2021
312	1	USH2A	c.5573-2A>G	–	Splicing	I27	–	European	Europe	Baux et al., 2014

No.	Number of patients	Phenotype	Nucleotide change[a]	Amino acid change[a]	Variant type[b]	Exon/ Intron[c]	Domain	Geographical/ Ethnic distribution	Continent	Reference
313	1	USH2A	c.5776+1G>A	–	Splicing	I28	–	European	Europe	Glöckle et al., 2014
314	1	USH2A	c.5776+1G>A	–	Splicing	I28	–	Kingdom of Saudi Arabia	Asia	Neuhaus et al., 2017
315	1	USH2A	c.5776+1G>A	–	Splicing	I28	–	Spanish	Europe	Fuster-García et al., 2018
316	2	USH2A	c.5777-1G>A	p.E1926_A1952 del	Splicing	I28	–	Newfound-land	America	Pater et al., 2019
317	1	USH2A	c.7452-1G>A	–	Splicing	I39	–	European	Europe	Bonnet et al., 2016
318	1	USH2A	c.8558+1G>T	–	Splicing	I42	–	Israeli	Asia	Khalaileh et al., 2018
319	1	RP	c.8559-2A>G	p.Y2854_R2894 del	Splicing	I42	–	Chinese	Asia	Chen et al., 2014
320	1	RP	c.8559-2A>G	–	Splicing	I42	–	Japanese	Asia	Zhao et al., 2014
321	3	USH2A	c.8559-2A>G	–	Splicing	I42	–	Chinese	Asia	Li et al., 2015
322	1	USH2A	c.8559-2A>G	–	Splicing	I42	–	Chinese	Asia	Jiang et al., 2015
323	3	USH2A	c.8559-2A>G	–	Splicing	I42	–	Chinese	Asia	Sun et al., 2018
324	2	RP	c.8559-2A>G	–	Splicing	I42	–	Japanese	Asia	Koyanagi et al., 2019
325	1	USH2A	c.8559-2A>G	–	Splicing	I42	–	Japanese	Asia	Inaba et al., 2020
326	2	USH2A	c.8559-2A>G	–	Splicing	I42	–	Chinese	Asia	Gao et al., 2021
327	3	USH2A	c.8559-2A>G	–	Splicing	I42	–	Chinese	Asia	Zhu et al., 2021
328	2	RP	c.8559-2A>G	–	Splicing	I42	–	Chinese	Asia	Zhu et al., 2021
329	1	USH2A	c.8559-2A>G	–	Splicing	I42	–	Chinese	Asia	Meng et al., 2021
330	1	USH2A	c.8682-9A>G	–	Splicing	I43	–	European	Europe	Hartel et al., 2016
331	1	RP	c.8682-9A>G	–	Splicing	I43	–	United States	America	Zampaglione et al., 2020
332	1	USH2A	c.8682-9A>G	–	Splicing	I43	–	–	–	Wafa et al., 2021
333	1	USH2A	c.9570+1G>A	–	Splicing	I48	–	Asian	Asia	Brodie et al., 2021
334	1	USH2A	c.11389+1G>A	–	Splicing	I58	–	Mexican	America	Zenteno et al., 2020
335	1	USH2A	c.11389+3A>T	–	Splicing	I58	–	–	–	Bahena et al., 2022
336	2	USH2A	c.12067-2A>G	–	Splicing	I61	–	Bukhara	Asia	Auslender et al., 2008
337	1	USH2A	c.12067-2A>G	–	Splicing	I61	–	United States	America	McGee et al., 2010
338	1	USH2A	c.12067-2A>G	–	Splicing	I61	–	Spanish	Europe	Garcia-Garcia et al., 2011
339	1	USH2A	c.12067-2A>G	–	Splicing	I61	–	European	Europe	Bonnet et al., 2016
340	1	USH2A	c.12067-2A>G	–	Splicing	I61	–	Jewish M-Asia	Asia	Neuhaus et al., 2017
341	7	USH2A	c.12067-2A>G	–	Splicing	I61	–	Israeli	Asia	Khalaileh et al., 2018

No.	Number of patients	Phenotype	Nucleotide change[a]	Amino acid change[a]	Variant type[b]	Exon/ Intron[c]	Domain	Geographical/ Ethnic distribution	Continent	Reference
342	1	USH2A	c.12067-2A>G	–	Splicing	I61	–	–	–	Bahena et al., 2022
343	1	USH2A	c.12067-1G>C	–	Splicing	I61	–	–	–	Bahena et al., 2022

RP, retinitis pigmentosa; USH2A, Usher syndrome type IIA; *USH2A*, the usherin gene; SP, signal peptide; Lam NT, laminin N-terminal; EGF Lam, laminin epidermal growth factor-like; FN III, fibronectin type-III; TM, transmembrane.

[a] The description of most variants is recalibrated following the Human Genome Variation Society nomenclature (https://varnomen.hgvs.org/) using the reference sequence (NG_009497.2, NM_206933.4).

[b] For the description of variant type:
(1) Frameshift includes small deletion and duplication/insertion with changes involving 20 bp or less leading to reading frame shift;
(2) Small indel includes duplication and deletion-insertion with changes involving 20 bp or less leading to one or more amino acids inserted or replaced;
(3) Gross deletion, referring to deletion over 20 bp, may be described at the genomic DNA level or cDNA level as reported in the reference articles;
(4) Complex rearrangement includes complex deletion-insertion.

[c] The number after "E" or "I" indicates the variant's location in exon (E) or intron (I) of the *USH2A* gene.

[#] Patient has two responsible *USH2A* homozygous variants.

2 Supplementary References

Abdi, S., Bahloul, A., Behlouli, A., Hardelin, J. P., Makrelouf, M., Boudjelida, K., et al. (2016). Diversity of the genes implicated in Algerian patients affected by Usher syndrome. *PLoS One* 11 (9), e0161893. doi:10.1371/journal.pone.0161893

Adato, A., Weston, M. D., Berry, A., Kimberling, W. J., and Bonne-Tamir, A. (2000). Three novel mutations and twelve polymorphisms identified in the USH2A gene in Israeli USH2 families. *Hum. Mutat*. 15 (4), 388. doi:10.1002/(SICI)1098-1004(200004)15:4<388::AID-HUMU27>3.0.CO;2-N

Ahmed, A. N., Tahir, R., Khan, N., Ahmad, M., Dawood, M., Basit, A., et al. (2021). USH2A gene variants cause Keratoconus and Usher syndrome phenotypes in Pakistani families. *BMC Ophthalmol.* 21 (1), 191. doi:10.1186/s12886-021-01957-9

Aller, E., Jaijo, T., Beneyto, M., Nájera, C., Oltra, S., Ayuso, C., et al. (2006). Identification of 14 novel mutations in the long isoform of USH2A in Spanish patients with Usher syndrome type II. *J. Med. Genet.* 43 (11), e55. doi:10.1136/jmg.2006.041764

Aller, E., Nájera, C., Millán, J. M., Oltra, J. S., Pérez-Garrigues, H., Vilela, C., et al. (2004). Genetic analysis of 2299delG and C759F mutations (USH2A) in patients with visual and/or auditory impairments. *Eur. J. Hum. Genet.* 12 (5), 407–410. doi:10.1038/sj.ejhg.5201138

Auslender, N., Bandah, D., Rizel, L., Behar, D. M., Shohat, M., Banin, E., et al. (2008). Four USH2A founder mutations underlie the majority of Usher syndrome type 2 cases among non-Ashkenazi Jews. *Genet. Test*. 12 (2), 289–294. doi:10.1089/gte.2007.0107

Ávila-Fernández, A., Cantalapiedra, D., Aller, E., Vallespín, E., Aguirre-Lambán, J., Blanco-Kelly, F., et al. (2010). Mutation analysis of 272 Spanish families affected by autosomal recessive retinitis pigmentosa using a genotyping microarray. *Mol. Vis*. 16, 2550–2558.

Bahena, P., Daftarian, N., Maroofian, R., Linares, P., Villalobos, D., Mirrahimi, M., et al. (2022). Unraveling the genetic complexities of

combined retinal dystrophy and hearing impairment. *Hum. Genet*. 141 (3-4), 785–803. doi:10.1007/s00439-021-02303-1

Baux, D., Blanchet, C., Hamel, C., Meunier, I., Larrieu, L., Faugère, V., et al. (2014). Enrichment of LOVD-USHbases with 152 USH2A genotypes defines an extensive mutational spectrum and highlights missense hotspots. *Hum. Mutat*. 35 (10), 1179–1186. doi:10.1002/humu.22608

Baux, D., Larrieu, L., Blanchet, C., Hamel, C., Ben Salah, S., Vielle, A., et al. (2007). Molecular and in silico analyses of the full-length isoform of usherin identify new pathogenic alleles in Usher type II patients. *Hum. Mutat*. 28 (8), 781–789. doi:10.1002/humu.20513

Bernal, S., Ayuso, C., Antiñolo, G., Gimenez, A., Borrego, S., Trujillo, M. J., et al. (2003). Mutations in USH2A in Spanish patients with autosomal recessive retinitis pigmentosa: High prevalence and phenotypic variation. *J. Med. Genet*. 40 (1), e8. doi:10.1136/jmg.40.1.e8

Bernal, S., Medà, C., Solans, T., Ayuso, C., Garcia-Sandoval, B., Valverde, D., et al. (2005). Clinical and genetic studies in Spanish patients with Usher syndrome type II: Description of new mutations and evidence for a lack of genotype--phenotype correlation. *Clin. Genet*. 68 (3), 204–214. doi:10.1111/j.1399-0004.2005.00481.x

Blanco-Kelly, F., Jaijo, T., Aller, E., Avila-Fernandez, A., López-Molina, M. I., Giménez, A., et al. (2015). Clinical aspects of Usher syndrome and the USH2A gene in a cohort of 433 patients. *JAMA Ophthalmol*. 133 (2), 157–164. doi:10.1001/jamaophthalmol.2014.4498

Bonnet, C., Riahi, Z., Chantot-Bastaraud, S., Smagghe, L., Letexier, M., Marcaillou, C., et al. (2016). An innovative strategy for the molecular diagnosis of Usher syndrome identifies causal biallelic mutations in 93% of European patients. *Eur. J. Hum. Genet*. 24 (12), 1730–1738. doi:10.1038/ejhg.2016.99

Brodie, K. D., Moore, A. T., Slavotinek, A. M., Meyer, A. K., Nadaraja, G. S., Conrad, D. E., et al. (2021). Genetic testing leading to early identification of childhood ocular manifestations of Usher syndrome. *Laryngoscope* 131 (6), E2053–E2059. doi:10.1002/lary.29193

Chen, X., Sheng, X., Liu, X., Li, H., Liu, Y., Rong, W., et al. (2014). Targeted next-generation sequencing reveals novel USH2A mutations associated with diverse disease phenotypes: Implications for clinical and molecular diagnosis. *PLoS One* 9 (8), e105439. doi:10.1371/journal.pone.0105439

Comander, J., Weigel-DiFranco, C., Maher, M., Place, E., Wan, A., Harper, S., et al. (2017). The genetic basis of pericentral retinitis pigmentosa-a form of mild retinitis pigmentosa. *Genes* 8 (10), 256. doi:10.3390/genes8100256

Coppieters, F., Van Schil, K., Bauwens, M., Verdin, H., De Jaegher, A., Syx, D., et al. (2014). Identity-by-descent-guided mutation analysis and exome sequencing in consanguineous families reveals unusual clinical and molecular findings in retinal dystrophy. *Genet. Med*. 16 (9), 671–680. doi:10.1038/gim.2014.24

Dad, S., Rendtorff, N. D., Tranebjærg, L., Grønskov, K., Karstensen, H. G., Brox, V., et al. (2016). Usher syndrome in Denmark: Mutation spectrum and some clinical observations. *Mol. Genet. Genomic Med*. 4 (5), 527–539. doi:10.1002/mgg3.228

Dan, H., Huang, X., Xing, Y., and Shen, Y. (2020). Application of targeted panel sequencing and whole exome sequencing for 76 Chinese families with retinitis pigmentosa. *Mol. Genet. Genomic Med*. 8 (3), e1131. doi:10.1002/mgg3.1131

de Castro-Miró, M., Pomares, E., Lorés-Motta, L., Tonda, R., Dopazo, J., Marfany, G., et al. (2014). Combined genetic and high-throughput strategies for molecular diagnosis of inherited retinal dystrophies. *PLoS One* 9 (2), e88410. doi:10.1371/journal.pone.0088410

Dreyer, B., Tranebjaerg, L., Rosenberg, T., Weston, M. D., Kimberling, W. J., Nilssen, O. (2000). Identification of novel USH2A mutations: Implications for the structure of USH2A protein. *Eur. J. Hum. Genet*. 8 (7), 500–506. doi:10.1038/sj.ejhg.5200491

DuPont, M., Jones, E. M., Xu, M., and Chen, R. (2018). Investigating the disease association of USH2A p.C759F variant by leveraging large retinitis pigmentosa cohort data. *Ophthalmic Genet*. 39 (2), 291–292. doi:10.1080/13816810.2017.1418388

Eandi, C. M., Dallorto, L., Spinetta, R., Micieli, M. P., Vanzetti, M., Mariottini, A., et al. (2017). Targeted next generation sequencing in Italian patients with Usher syndrome: Phenotype-genotype correlations. *Sci. Rep*. 7 (1), 15681. doi:10.1038/s41598-017-16014-z

Ebermann, I., Koenekoop, R. K., Lopez, I., Bou-Khzam, L., Pigeon, R., and Bolz, H. J. (2009). An USH2A founder mutation is the major cause of Usher syndrome type 2 in Canadians of French origin and confirms common roots of Quebecois and Acadians. *Eur. J Hum. Genet*. 17 (1), 80–84. doi:10.1038/ejhg.2008.143

Ebermann, I., Phillips, J. B., Liebau, M. C., Koenekoop, R. K., Schermer, B., Lopez, I., et al. (2010). PDZD7 is a modifier of retinal disease and a contributor to digenic Usher syndrome. *J. Clin. Invest*. 120 (6), 1812–1823. doi:10.1172/JCI39715

Eudy, J. D., Weston, M. D., Yao, S., Hoover, D. M., Rehm, H. L., Ma-Edmonds, M., et al. (1998). Mutation of a gene encoding a protein with extracellular matrix motifs in Usher syndrome type IIa. *Science* 280 (5370), 1753–1757. doi:10.1126/science.280.5370.1753

Falsini, B., Placidi, G., De Siena, E., Savastano, M. C., Minnella, A. M., Maceroni, M., et al. (2021). USH2A-related retinitis pigmentosa: Staging of disease severity and morpho-functional studies. *Diagnostics* 11 (2), 213. doi:10.3390/diagnostics11020213

Fu, J., Shen, S., Cheng, J., Lv, H., and Fu, J. (2020). A case of Usher syndrome type IIA caused by a rare USH2A homozygous frameshift variant with maternal uniparental disomy (UPD) in a Chinese family. *J. Cell. Mol. Med*. 24 (14), 7743–7750. doi:10.1111/jcmm.15405

Fuster-García, C., García-García, G., Jaijo, T., Fornés, N., Ayuso, C., Fernández-Burriel, M., et al. (2018). High-throughput sequencing for the molecular diagnosis of Usher syndrome reveals 42 novel mutations and consolidates CEP250 as Usher-like disease causative. *Sci. Rep*. 8 (1), 17113. doi:10.1038/s41598-018-35085-0

Gao, F. J., Wang, D. D., Chen, F., Sun, H. X., Hu, F. Y., Xu, P., et al. (2021). Prevalence and genetic-phenotypic characteristics of patients with USH2A mutations in a large cohort of Chinese patients with inherited retinal disease. *Br. J. Ophthalmol*. 105 (1), 87–92. doi:10.1136/bjophthalmol-2020-315878

García Bohórquez, B., Aller, E., Rodríguez Muñoz, A., Jaijo, T., García García, G., and Millán, J. M. (2021). Updating the genetic landscape of inherited retinal dystrophies. *Front. Cell Dev. Biol*. 9, 645600. doi:10.3389/fcell.2021.645600

Garcia-Garcia, G., Aparisi, M. J., Jaijo, T., Rodrigo, R., Leon, A. M., Avila-Fernandez, A., et al. (2011). Mutational screening of the USH2A gene in Spanish USH patients reveals 23 novel pathogenic mutations. *Orphanet J. Rare Dis*. 6, 65. doi:10.1186/1750-1172-6-65

Glöckle, N., Kohl, S., Mohr, J., Scheurenbrand, T., Sprecher, A., Weisschuh, N., et al. (2014). Panel-based next generation sequencing as a reliable and efficient technique to detect mutations in unselected patients with retinal dystrophies. *Eur. J. Hum. Genet*. 22 (1), 99–104. doi:10.1038/ejhg.2013.72

Hartel, B. P., Löfgren, M., Huygen, P. L., Guchelaar, I., Lo-A-Njoe Kort, N., Sadeghi, A. M., et al. (2016). A combination of two truncating mutations in USH2A causes more severe and progressive hearing impairment in Usher syndrome type IIa. *Hear Res*. 339, 60–68. doi:10.1016/j.heares.2016.06.008

Hartel, B. P., van Nierop, J. W. I., Huinck, W. J., Rotteveel, L. J. C., Mylanus, E. A. M., Snik, A. F., et al. (2017). Cochlear implantation in patients with Usher syndrome type IIa increases performance and quality of life. *Otol. Neurotol*. 38 (6), e120–e127. doi:10.1097/MAO.0000000000001441

Inaba, A., Maeda, A., Yoshida, A., Kawai, K., Hirami, Y., Kurimoto, Y., et al. (2020). Truncating variants contribute to hearing loss and severe retinopathy in USH2A-associated retinitis pigmentosa in Japanese patients. *Int. J. Mol. Sci*. 21 (21), 7817. doi:10.3390/ijms21217817

Jaijo, T., Aller, E., García-García, G., Aparisi, M. J., Bernal, S., Avila-Fernández, A., et al. (2010). Microarray-based mutation analysis of 183 Spanish families with Usher syndrome. *Invest. Ophthalmol. Vis. Sci*. 51 (3), 1311–1317. doi:10.1167/iovs.09-4085

Jiang, L., Liang, X., Li, Y., Wang, J., Zaneveld, J. E., Wang, H., et al. (2015). Comprehensive molecular diagnosis of 67 Chinese Usher syndrome probands: High rate of ethnicity specific mutations in Chinese USH patients. *Orphanet J. Rare Dis*. 10, 110. doi:10.1186/s13023-015-0329-3

Kaiserman, N., Obolensky, A., Banin, E., and Sharon, D. (2007). Novel USH2A mutations in Israeli patients with retinitis pigmentosa and Usher syndrome type 2. *Arch. Ophthalmol*. 125 (2), 219–224. doi:10.1001/archopht.125.2.219

Karali, M., Testa, F., Brunetti-Pierri, R., Di Iorio, V., Pizzo, M., Melillo, P., et al. (2019). Clinical and genetic analysis of a European cohort with pericentral retinitis pigmentosa. *Int. J. Mol. Sci*. 21 (1), 86. doi:10.3390/ijms21010086

Katagiri, S., Akahori, M., Sergeev, Y., Yoshitake, K., Ikeo, K., Furuno, M., et al. (2014). Whole exome analysis identifies frequent CNGA1 mutations in Japanese population with autosomal recessive retinitis pigmentosa. *PLoS One* 9 (9), e108721. doi:10.1371/journal.pone.0108721

Khalaileh, A., Abu-Diab, A., Ben-Yosef, T., Raas-Rothschild, A., Lerer, I., Alswaiti, Y., et al. (2018). The genetics of Usher syndrome in the Israeli and Palestinian populations. *Invest. Ophthalmol. Vis. Sci*. 59 (2), 1095–1104. doi:10.1167/iovs.17-22817

Koyanagi, Y., Akiyama, M., Nishiguchi, K. M., Momozawa, Y., Kamatani, Y., Takata, S., et al. (2019). Genetic characteristics of retinitis pigmentosa in 1204 Japanese patients. *J. Med. Genet*. 56 (10), 662–670. doi:10.1136/jmedgenet-2018-105691

Koyanagi, Y., Ueno, S., Ito, Y., Kominami, T., Komori, S., Akiyama, M., et al. (2020). Relationship between macular curvature and common causative genes of retinitis pigmentosa in Japanese patients. *Invest. Ophthalmol. Vis. Sci.* 61 (10), 6. doi:10.1167/iovs.61.10.6

Krawitz, P. M., Schiska, D., Krüger, U., Appelt, S., Heinrich, V., Parkhomchuk, D., et al. (2014). Screening for single nucleotide variants, small indels and exon deletions with a next-generation sequencing based gene panel approach for Usher syndrome. *Mol. Genet. Genomic Med. 2* (5). 393–401. doi:10.1002/mgg3.92

Leijendeckers, J. M., Pennings, R. J., Snik, A. F., Bosman, A. J., and Cremers, C. W. (2009). Audiometric characteristics of USH2a patients. *Audiol. Neurootol.* 14 (4), 223–231. doi:10.1159/000189265

Lenarduzzi, S., Vozzi, D., Morgan, A., Rubinato, E., D'Eustacchio, A., Osland, T. M., et al. (2015). Usher syndrome: An effective sequencing approach to establish a genetic and clinical diagnosis. *Hear Res*. 320, 18–23. doi:10.1016/j.heares.2014.12.006

Lenassi, E., Saihan, Z., Bitner-Glindzicz, M., and Webster, A. R. (2014). The effect of the common c.2299delG mutation in USH2A on RNA splicing. *Exp. Eye Res*. 122, 9–12. doi:10.1016/j.exer.2014.02.018

Lenassi, E., Vincent, A., Li, Z., Saihan, Z., Coffey, A. J., Steele-Stallard, H. B., et al. (2015). A detailed clinical and molecular survey of subjects with nonsyndromic USH2A retinopathy reveals an allelic hierarchy of disease-causing variants. *Eur. J. Hum. Genet*. 23 (10), 1318–1327. doi:10.1038/ejhg.2014.283

Le Quesne Stabej, P., Saihan, Z., Rangesh, N., Steele-Stallard, H. B., Ambrose, J., Coffey, A., et al. (2012). Comprehensive sequence analysis of nine Usher syndrome genes in the UK national collaborative Usher study. *J. Med. Genet.* 49 (1). 27–36. doi:10.1136/jmedgenet-2011-100468

Leroy, B. P., Aragon-Martin, J. A., Weston, M. D., Bessant, D. A., Willis, C., Webster, A. R., et al. (2001). Spectrum of mutations in USH2A in British patients with Usher syndrome type II. *Exp. Eye Res*. 72 (5), 503–509. doi:10.1006/exer.2000.0978

Li, P., Liu, F., Zhang, M., Wang, Q., and Liu, M. (2015). Analysis of USH2A gene mutation in a Chinese family affected with Usher syndrome. *Zhonghua Yi Xue Yi Chuan Xue Za Zhi* 32 (4), 468–471. doi:10.3760/cma.j.issn.1003-9406.2015.04.003

Liu, X., Tang, Z., Li, C., Yang, K., Gan, G., Zhang, Z., et al. (2010). Novel USH2A compound heterozygous mutations cause RP/USH2 in a Chinese family. *Mol. Vis*. 16, 454–461. doi:10.1167/3.9.454

Maubaret, C., Griffoin, J. M., Arnaud, B., and Hamel, C. (2005). Novel mutations in MYO7A and USH2A in Usher syndrome. *Ophthalmic Genet*. 26 (1), 25–29. doi:10.1080/13816810590918118

McGee, T. L., Seyedahmadi, B. J., Sweeney, M. O., Dryja, T. P., and Berson, E. L. (2010). Novel mutations in the long isoform of the USH2A gene in patients with Usher syndrome type II or non-syndromic retinitis pigmentosa. *J. Med. Genet*. 47 (7), 499–506. doi:10.1136/jmg.2009.075143

Meng, X., Liu, X., Li, Y., Guo, T., and Yang, L. (2021). Correlation between genotype and phenotype in 69 Chinese patients with USH2A mutations: A comparative study of the patients with Usher syndrome and nonsyndromic retinitis pigmentosa. *Acta Ophthalmol*. 99 (4), e447–e460. doi:10.1111/aos.14626

Molina-Ramírez, L. P., Lenassi, E., Ellingford, J. M., Sergouniotis, P. I., Ramsden, S. C., Bruce, I. A., et al. (2020). Establishing genotype-phenotype correlation in USH2A-related disorders to personalize audiological surveillance and rehabilitation. *Otol. Neurotol*. 41 (4), 431–437. doi:10.1097/MAO.0000000000002588

Nájera, C., Beneyto, M., Blanca, J., Aller, E., Fontcuberta, A., Millán, J. M., et al. (2002). Mutations in myosin VIIA (MYO7A) and usherin (USH2A) in Spanish patients with Usher syndrome types I and II, respectively. *Hum. Mutat*. 20 (1), 76–77. doi:10.1002/humu.9042

Nakanishi, H., Ohtsubo, M., Iwasaki, S., Hotta, Y., Usami, S., Mizuta, K., et al. (2011). Novel USH2A mutations in Japanese Usher syndrome type 2 patients: Marked differences in the mutation spectrum between the Japanese and other populations. *J Hum. Genet*. 56 (7), 484–490. doi:10.1038/jhg.2011.45

Neuhaus, C., Eisenberger, T., Decker, C., Nagl, S., Blank, C., Pfister, M., et al. (2017). Next-generation sequencing reveals the mutational landscape of clinically diagnosed Usher syndrome: Copy number variations, phenocopies, a predominant target for translational read-through, and PEX26 mutated in Heimler syndrome. *Mol. Genet. Genomic Med*. 5 (5), 531–552. doi:10.1002/mgg3.312

Oishi, M., Oishi, A., Gotoh, N., Ogino, K., Higasa, K., Iida, K., et al. (2014). Comprehensive molecular diagnosis of a large cohort of Japanese retinitis pigmentosa and Usher syndrome patients by next-generation sequencing. *Invest. Ophthalmol. Vis. Sci.* 55 (11), 7369–7375.

doi:10.1167/iovs.14-15458
Ouyang, X. M., Hejtmancik, J. F., Jacobson, S. G., Li, A. R., Du, L. L., Angeli, S., et al. (2004). Mutational spectrum in Usher syndrome type II. *Clin. Genet*. 65 (4), 288–293. doi:10.1046/j.1399-0004.2004.00216.x
Pater, J. A., Green, J., O'Rielly, D. D., Griffin, A., Squires, J., Burt, T., et al. (2019). Novel Usher syndrome pathogenic variants identified in cases with hearing and vision loss. *BMC Med. Genet*. 20 (1), 68. doi:10.1186/s12881-019-0777-z
Pennings, R. J., Huygen, P. L., Orten, D. J., Wagenaar, M., van Aarem, A., Kremer, H., et al. (2004a). Evaluation of visual impairment in Usher syndrome 1b and Usher syndrome 2a. *Acta Ophthalmol. Scand*. 82 (2), 131–139. doi:10.1111/j.1600-0420.2004.00234.x
Pennings, R. J., Huygen, P. L., Weston, M. D., van Aarem, A., Wagenaar, M., Kimberling, W. J., et al. (2003). Pure tone hearing thresholds and speech recognition scores in Dutch patients carrying mutations in the USH2A gene. *Otol. Neurotol*. 24 (1), 58–63. doi:10.1097/00129492-200301000-00013
Pennings, R. J., Te Brinke, H., Weston, M. D., Claassen, A., Orten, D. J., Weekamp, H., et al. (2004b). USH2A mutation analysis in 70 Dutch families with Usher syndrome type II. *Hum. Mutat*. 24 (2), 185. doi:10.1002/humu.9259
Pérez-Carro, R., Blanco-Kelly, F., Galbis-Martínez, L., García-García, G., Aller, E., García-Sandoval, B., et al. (2018). Unravelling the pathogenic role and genotype-phenotype correlation of the USH2A p.(Cys759Phe) variant among Spanish families. *PLoS One* 13 (6), e0199048. doi:10.1371/journal.pone.0199048
Perez-Carro, R., Corton, M., Sánchez-Navarro, I., Zurita, O., Sanchez-Bolivar, N., Sánchez-Alcudia, R., et al. (2016). Panel-based NGS reveals novel pathogenic mutations in autosomal recessive retinitis pigmentosa. *Sci. Rep*. 6, 19531. doi:10.1038/srep19531
Reddy, R., Fahiminiya, S., El Zir, E., Mansour, A., Megarbane, A., Majewski, J., et al. (2014). Molecular genetics of the Usher syndrome in Lebanon: Identification of 11 novel protein truncating mutations by whole exome sequencing. *PLoS One* 9 (9), e107326. doi:10.1371/journal.pone.0107326
Rivolta, C., Berson, E. L., and Dryja, T. P. (2002). Paternal uniparental heterodisomy with partial isodisomy of chromosome 1 in a patient with retinitis pigmentosa without hearing loss and a missense mutation in the Usher syndrome type II gene USH2A. *Arch. Ophthalmol*. 120 (11), 1566–1571. doi:10.1001/archopht.120.11.1566
Sadeghi, A. M., Cohn, E. S., Kimberling, W. J., Halvarsson, G., and Möller, C. (2013). Expressivity of hearing loss in cases with Usher syndrome type IIA. *Int. J. Audiol*. 52 (12), 832–837. doi:10.3109/14992027.2013.839885
Salmaninejad, A., Bedoni, N., Ravesh, Z., Quinodoz, M., Shoeibi, N., Mojarrad, M., et al. (2020). Whole exome sequencing and homozygosity mapping reveals genetic defects in consanguineous Iranian families with inherited retinal dystrophies. *Sci. Rep*. 10 (1), 19413. doi:10.1038/s41598-020-75841-9
Sandberg, M. A., Rosner, B., Weigel-DiFranco, C., McGee, T. L., Dryja, T. P., and Berson, E. L. (2008). Disease course in patients with autosomal recessive retinitis pigmentosa due to the USH2A gene. *Invest. Ophthalmol. Vis. Sci.* 49 (12). 5532–5539. doi:10.1167/iovs.08-2009
Santana, E. E., Fuster-García, C., Aller, E., Jaijo, T., García-Bohórquez, B., García-García, G., et al. (2019). Genetic screening of the Usher syndrome in Cuba. *Front. Genet*. 10, 501. doi:10.3389/fgene.2019.00501

Sengillo, J. D., Cabral, T., Schuerch, K., Duong, J., Lee, W., Boudreault, K., et al. (2017). Electroretinography reveals difference in cone function between syndromic and nonsyndromic USH2A patients. *Sci. Rep*. 7 (1), 11170. doi:10.1038/s41598-017-11679-y

Seyedahmadi, B. J., Rivolta, C., Keene, J. A., Berson, E. L., and Dryja, T. P. (2004). Comprehensive screening of the USH2A gene in Usher syndrome type II and non-syndromic recessive retinitis pigmentosa. *Exp. Eye Res*. 79 (2), 167–173. doi:10.1016/j.exer.2004.03.005

Shu, H. R., Bi, H., Pan, Y. C., Xu, H. Y., Song, J. X., and Hu, J. (2015). Targeted exome sequencing reveals novel USH2A mutations in Chinese patients with simplex Usher syndrome. *BMC Med. Genet*. 16, 83. doi:10.1186/s12881-015-0223-9

Sodi, A., Mariottini, A., Passerini, I., Murro, V., Tachyla, I., Bianchi, B., et al. (2014). MYO7A and USH2A gene sequence variants in Italian patients with Usher syndrome. *Mol. Vis*. 20, 1717–1731

Sun, T., Xu, K., Ren, Y., Xie, Y., Zhang, X., Tian, L., et al. (2018). Comprehensive molecular screening in Chinese Usher syndrome patients. *Invest. Ophthalmol. Vis. Sci*. 59 (3), 1229–1237. doi:10.1167/iovs.17-23312

Sun, Y., Li, W., Li, J. K., Wang, Z. S., Bai, J. Y., Xu, L., et al. (2020). Genetic and clinical findings of panel-based targeted exome sequencing in a northeast Chinese cohort with retinitis pigmentosa. *Mol. Genet. Genomic Med*. 8 (4), e1184. doi:10.1002/mgg3.1184

Toms, M., Dubis, A. M., de Vrieze, E., Tracey-White, D., Mitsios, A., Hayes, M., et al. (2020). Clinical and preclinical therapeutic outcome metrics for USH2A-related disease. *Hum. Mol. Genet*. 29 (11), 1882–1899. doi:10.1093/hmg/ddaa004

Wafa, T. T., Faridi, R., King, K. A., Zalewski, C., Yousaf, R., Schultz, J. M., et al. (2021). Vestibular phenotype-genotype correlation in a cohort of 90 patients with Usher syndrome. *Clin. Genet*. 99 (2), 226–235. doi:10.1111/cge.13868

Watson, C. M., El-Asrag, M., Parry, D. A., Morgan, J. E., Logan, C. V., Carr, I. M., et al. (2014). Mutation screening of retinal dystrophy patients by targeted capture from tagged pooled DNAs and next generation sequencing. *PLoS One* 9 (8), e104281. doi:10.1371/journal.pone.0104281

Xing, D., Zhou, H., Yu, R., Wang, L., Hu, L., Li, Z., et al. (2020). Targeted exome sequencing identified a novel USH2A mutation in a Chinese usher syndrome family: A case report. *BMC Ophthalmol.* 20 (1), 485. doi:10.1186/s12886-020-01711-7

Yan, D., Ouyang, X., Patterson, D. M., Du, L. L., Jacobson, S. G., and Liu, X. Z. (2009). Mutation analysis in the long isoform of USH2A in American patients with Usher Syndrome type II. *J. Hum. Genet*. 54 (12), 732–738. doi:10.1038/jhg.2009.107

Zampaglione, E., Kinde, B., Place, E. M., Navarro-Gomez, D., Maher, M., Jamshidi, F., et al. (2020). Copy-number variation contributes 9% of pathogenicity in the inherited retinal degenerations. *Genet. Med*. 22 (6), 1079–1087. doi:10.1038/s41436-020-0759-8

Zenteno, J. C., García-Montaño, L. A., Cruz-Aguilar, M., Ronquillo, J., Rodas-Serrano, A., Aguilar-Castul, L., et al. (2020). Extensive genic and allelic heterogeneity underlying inherited retinal dystrophies in Mexican patients molecularly analyzed by next-generation sequencing. *Mol. Genet. Genomic Med.* 8 (1), 10.1002/mgg3.1044. doi:10.1002/mgg3.1044

Zhao, Y., Hosono, K., Suto, K., Ishigami, C., Arai, Y., Hikoya, A., et al. (2014). The first USH2A mutation analysis of Japanese autosomal recessive retinitis pigmentosa patients: A totally different mutation profile with the lack of frequent mutations found in Caucasian patients. *J. Hum. Genet*. 59 (9), 521–528. doi:10.1038/jhg.2014.65

Zhu, T., Chen, D. F., Wang, L., Wu, S., Wei, X., Li, H., et al. (2021). USH2A variants in Chinese patients with Usher syndrome type II and non-syndromic retinitis pigmentosa. *Br. J. Ophthalmol*. 105 (5), 694–703. doi:10.1136/bjophthalmol-2019-315786

Zupan, A., Fakin, A., Battelino, S., Jarc-Vidmar, M., Hawlina, M., Bonnet, C., et al. (2019). Clinical and haplotypic variability of Slovenian USH2A patients homozygous for the c. 11864G>A nonsense mutation. *Genes* 10 (12), 1015. doi:10.3390/genes10121015
